# Epidermal growth factor receptor inhibition potentiates chemotherapeutics‐mediated sensitization of metastatic breast cancer stem cells

**DOI:** 10.1002/cnr2.2049

**Published:** 2024-03-24

**Authors:** Trisha Kar, Prachi Dugam, Surbhi Shivhare, Swathi R. Shetty, Subholakshmi Choudhury, Debanjan Sen, Barnali Deb, Swapan Majumdar, Sudhan Debnath, Amitava Das

**Affiliations:** ^1^ Department of Applied Biology Council of Scientific and Industrial Research‐Indian Institute of Chemical Technology (CSIR‐IICT) Hyderabad Telangana India; ^2^ Academy of Scientific and Innovative Research (AcSIR) Ghaziabad India; ^3^ Department of Pharmaceutical Chemistry BCDA College of Pharmacy and Technology Kolkata West Bengal India; ^4^ Department of Chemistry Tripura University Agartala Tripura India; ^5^ Department of Chemistry Netaji Subhash Mahavidyalaya Udaipur Tripura India

**Keywords:** breast cancer, chemosensitization, EGFR, EGFR inhibitors, orthotopic xenotransplantation

## Abstract

**Background:**

Metastasis has been a cause of the poor prognosis and cancer relapse of triple‐negative breast cancer (TNBC) patients. The metastatic nature of TNBC is contributed by the breast cancer stem cells (CSCs) which have been implicated in tumorigenesis. Higher expression of epidermal growth factor receptor (EGFR) in breast CSCs has been used as a molecular target for breast cancer therapeutics. Thus, it necessitates the design and generation of efficacious EGFR inhibitors to target the downstream signaling associated with the cellular proliferation and tumorigenesis of breast cancer.

**Aim:**

To generate efficacious EGFR inhibitors that can potentiate the chemotherapeutic‐mediated mitigation of breast cancer tumorigenesis.

**Methods and Results:**

We identified small molecule EGFR inhibitors using molecular docking studies. In‐vitro screening of the compounds was undertaken to identify the cytotoxicity profile of the small‐molecule EGFR inhibitors followed by evaluation of the non‐cytotoxic compounds in modulating the doxorubicin‐induced migration, in‐vitro tumorigenesis potential, and their effect on the pro‐apoptotic genes' and protein markers' expression in TNBC cells. Compound **1e** potentiated the doxorubicin‐mediated inhibitory effect on proliferation, migration, in‐vitro tumorigenesis capacity, and induction of apoptosis in *MDA‐MB‐231* cells, and in the sorted CD24^+^‐breast cancer cells and CD24^−^/CD44^+^‐breast CSC populations. Orthotopic xenotransplantation of the breast CSCs‐induced tumors in *C57BL/6J* mice was significantly inhibited by the low dose of Doxorubicin in the presence of compound **1e** as depicted by molecular and immunohistochemical analysis.

**Conclusion:**

Thus, the study suggests that EGFR inhibition‐mediated sensitization of the aggressive and metastatic breast CSCs in TNBCs toward chemotherapeutics may reduce the relapse of the disease.

## 1 INTRODUCTION

Breast cancer, among all types of cancers, is the second major cause of mortality in women. It represents 18.46% of all cancers leading to high mortality among women.^1^ Unlike other subtypes of breast cancer, triple‐negative breast cancer cells (TNBCs) lack the expression of hormonal receptors such as Estrogen receptor (ER), Progesterone receptor (PR), and Human epidermal growth factor receptor 2 (HER2) resulting in difficulty in targeting with hormonal receptor‐targeted therapeutics. The aggressive nature of TNBCs is attributed to the presence of cancer stem cells (CSCs).^2^ The CSCs are the tumor‐initiating cells that possess self‐renewal properties and can undergo asymmetric cell division, and differentiation.^3^ These CSCs are highly chemo‐ and radio‐resistant.^3^ Breast CSCs usually express unique cell surface markers referred to as Clusters of Differentiation (CD) and are characterized by the absence or low levels of CD24 and high expression levels of CD44 (CD24^−^/CD44^+^), which are frequently utilized to isolate and characterize breast CSCs from cancer cells to understand disease pathology and development of effective anticancer therapeutics.^3^ These breast CSCs are highly migratory and invade distant organs like the lungs, liver, bone, and brain by the phenomenon known as metastasis causing the relapse of cancer.^4^ Breast CSCs express different kinds of receptor tyrosine kinases (RTKs) like Platelet‐derived growth factor receptor β that renders them highly metastatic.^5^ TNBCs, the basal subtype of breast cancer, which is highly metastatic due to the presence of CD24^−^/CD44^+^‐breast CSCs, often express epidermal growth factor receptor (EGFR) in high levels, which serve as a biomarker.^6^ Our prior studies identified that the structural modification of a natural product Chrysin, into an EGFR inhibitor, effectively induced mesenchymal‐to‐epithelial transition in the metastatic breast CSCs and rendered them chemo‐sensitized toward chemotherapeutics.^6^ The erythroblastic leukemia viral oncogene (ErbB) family of tyrosine kinase includes four receptors—EGFR/ErbB1, HER2/ErbB2, HER3/ErbB3, and HER4/ErbB4. The binding of ligands such as epidermal growth factor (EGF), transforming growth factor‐alpha (TGF‐α), epigen, betacellulin, and amphiregulin bind to EGFR, initiate receptor dimerization and tyrosine auto‐phosphorylation, triggering multiple cellular signaling cascades which aid in the proliferation, growth, adhesion, differentiation, migration, and survival of breast cancer cells.^7^ The activation of EGFR in turn phosphorylates/activates its downstream signaling molecules Akt, ERK‐1/2, JNK, and p38 which contribute to the tumorigenesis and metastasis of cancer cells.^8^ Thus, suggesting EGFR as a molecular therapeutic target against breast cancer tumorigenesis. However, the use of small molecule EGFR tyrosine kinase inhibitors (TKIs) alone has shown minimal clinical effectiveness though with reduced side effects as compared with conventional chemotherapeutic drugs in breast cancer patients.^9^ Although recent advancements in the development of anti‐EGFR compounds have shown partial efficacy in mitigating breast cancer metastasis still there exist limitations due to the mutations in the EGFR gene resulting in resistance to these inhibitors.^10^, ^11^ Activation of bypass signaling of EGFR through alternative oncogenic pathways, phenotypic transformations, and fusions of carcinogenic genes also contribute to this resistance mechanism of EGFR‐TKIs.^12^ A small molecule compound S62, which inhibits the PELI1/EGFR interaction has depicted an effective strategy for inhibiting breast cancer metastasis.^13^ Increased phosphorylation of EGFR T654 residue leads to increased EMT, migration, invasion, and metastasis.^14^ The EGFR inhibitor Mitogen Induced Gene‐6 (Mig6) has prevented apoptosis, resulting in the inhibition of metastasis in TNBC *MDA‐MB‐231* cells.^15^ TIEG1 significantly inhibits the invasion of breast cancer cells, suppresses tumorigenesis in mice xenografts, and decreases metastasis to the lungs by inhibiting transcription of the EGFR gene and the EGFR signaling pathway.^16^ These studies suggest the crucial role of EGFR in potentiating the tumorigenesis and metastasis of TNBCs.

This implies the need for potent small‐molecule EGFR inhibitors effectively mitigating metastatic breast CSCs‐induced tumorigenesis. The present study investigated the efficacy of EGFR inhibiting compound **1e**, using in‐vitro and in‐vivo methods, in potentiating doxorubicin‐mediated reduced tumorigenesis by sensitization of the breast CSCs toward chemotherapeutic to prevent the relapse of cancer.

## 2 MATERIALS AND METHODS

### 2.1 Ligand dataset, protein source, and computational calculations

The x‐ray crystal structure of target receptor EGFR tyrosine kinase, complexed with co‐ligand 2a (3W32, 1.80 Å)^17^, ^18^ was retrieved from the RCSB Protein Data Bank. The protein's crystal structure was imported in AutoDock Tools 1.5.6 (ADT). The water molecules and hetero atoms were removed, and then polar hydrogen was added in the ADT interface, followed by the addition of computing Gasteiger and Kollman's charge. Finally, the proteins were saved in pdb format and utilized for molecular docking studies. The molecular docking was performed with Windows 10, OS architecture 64‐bit, Core (TM) 2 Due CPU machine. The protein‐ligand complex interaction was visualized using PyMOL (The PyMOL Molecular Graphics System, Version 2.0, Schrodinger, LLC) and ProteinPlus.^19^, ^20^

### 2.2 Molecular docking of synthesized inhibitors on EGFR

The molecular docking approach has been widely used to discover a bioactive molecule against a particular target. In the current study, the non‐commercial softwares‐Autodock Vina^21^ and Autodock 4.2^22^ were used for performing the molecular docking. The binding energy of the designed and synthesized compounds as small molecule EGFR inhibitors^23^ within the active site of EGFR tyrosine kinase was predicted. Preparation of the grid box was carried out by selecting the active site residues of EGFR tyrosine kinase such as ALA‐743, LYS‐745, CYS‐775, ARG‐776, LEU‐777, LEU‐788, THR‐790, GLN‐791, LEU‐792, MET‐793, GLY‐796, ARG‐841, ASN‐842, LEU‐844, THR‐854, ASP‐855, PHE‐856, and LEU‐858 after importing it in ADT. The active site interacting amino acid residues were identified from a 2D interaction diagram of the X‐ray crystal structure. The coordinate of the grid box center for 3 W32 was *x* = 17, *y* = 34, *z* = 14 while that of the grid box size dimension was *x* = 68, *y* = 60, *z* = 60. The docking was performed in AMDock: Assisted Molecular Docking with Autodock4 and Autodock Vina version 1.5.^24^ The force field used for docking using Autodock was Amber, with exhaustiveness and the number of runs was observed to be 25. Similarly, the force field for Autodock Vina used Charmm and exhaustiveness was 25.

### 2.3 Validation of docking protocol

Validation of the docking methodology is an important criterion for the accuracy of docking, and it was measured by calculating the root mean square deviation (RMSD). The RMSD value was calculated by superimposing the docked conformation of the co‐ligand on its original crystallographic bound conformation. A low value of RMSD indicates the high accuracy of the docking process. Depending on the ligand size, the RMSD values less than 1.50 or 2.0 Å were regarded as a better performance.^25^

### 2.4 Cell culture studies

TNBC cell lines (ER^−^, PR^−^, HER2^−^)—*MDA‐MB‐231* (ATCC HTB‐26) and *MDA‐MB‐468* (ATCC HTB‐132) were procured from ATCC and cultured in Roswell Park Memorial Institute Media (*RPMI‐1640*) and Dulbecco's Modified Eagle's Medium (DMEM), respectively, supplemented with 10% FBS and 1% Penicillin–Streptomycin solution. Cells were maintained at 5% CO_2_ at 37°C in a humid atmosphere.^6^, ^26^ The control cell line, Human Embryonic Kidney—*HEK‐293* (ATCC CRL‐3216) was also obtained from ATCC and cultured in DMEM.^5^, ^25^

### 2.5 Cytotoxicity studies

To evaluate the cytotoxicity profile of the potential EGFR inhibitors (compounds) against TNBC cell lines—*MDA‐MB‐231*, *MDA‐MB‐468*, and control cell line *HEK293*, cells were seeded at a cell density of 5 × 10^3^ cells/well in complete medium onto 96‐well plates. Here, the non‐tumoral human embryonic kidney (HEK293) epithelial cells have been utilized as a control cell line to compare the IC50 values of the compounds between cancerous and non‐cancerous cell lines with a big therapeutic window. The use of HEK293 cells as compared to other non‐cancerous cell lines are easy to maintain in culture and the results obtained from *HEK293* are highly reproducible and consistent.^27^ Briefly, the cells were incubated at 37°C for 12 h to attain morphology. After 12 h, cells were serum starved by replacing the complete media with media containing 2% FBS to synchronize the cells. Post‐12 h of synchronization, cells were treated with an increasing concentration (0.1, 1, 10, 100 μM) of all the compounds (1a, 1b, 1c, 1d, 1e, 1f, 1g, 1h, 1i, 1j, 1k). Separately, TNBC cells were treated with increasing concentration (0.01, 0.1, 1 μM) of Doxorubicin (Sigma‐Aldrich, St Louis, MO, USA) in presence of the selected compounds (1c, 1e, 1g, 1h, 1j, and 1k) at 5 μM concentration or combination of selected compounds (1c, 1e, 1g, 1h, 1j, and 1k) at increasing concentration (1, 2.5, 5, 10 μM) in presence of Doxorubicin (0.01 μM) for 48 h. In addition, the CD24^+^‐breast cancer cells and CD24^−^/CD44^+^‐breast CSC populations, being sorted by Magnetic Activated Cell Sorting (MACS) from *MDA‐MB‐231* cells as described previously,^6^ were also treated with increasing concentrations of Doxorubicin (0.01–1 μM), in the presence or absence of **1e** (10–30 μM), or PD153035 (10 μM) (Sigma‐Aldrich, St Louis, MO, USA). MTT assay was performed to evaluate the cytotoxicity of these compounds against the TNBC cell lines, and the sorted populations as described previously.^6^ All the experiments were performed thrice and the IC50 values of the compounds and Doxorubicin were calculated using GraphPad Prism software version 9 (San Diego, CA, USA). Similarly, the percent inhibition of the sorted cell populations by chemotherapeutic drug, doxorubicin in the absence/presence of potent compound **1e** or standard EGFR inhibitor, PD153035 was evaluated utilizing GraphPad Prism Version 9 (San Diego, CA, USA).

### 2.6 Cell migration (chemotaxis)

TNBC cell lines—*MDA‐MB‐231*, *MDA‐MB‐468*, and the sorted CD24^+^‐breast cancer cells, and CD24^−^/CD44^+^‐breast CSC populations from *MDA‐MB‐231* ^6^ were seeded at a density of 1 × 10^6^ cells/well to evaluate their migratory potential by Boyden‐Chamber assay. Post‐24 h serum starvation, the cells were subjected to treatment with the potential EGFR inhibitors (compounds—1c, 1e, 1g, 1h, 1j, and 1k) (5 μM) (based on their low IC50 values) or a commercial EGFR inhibitor, PD153035 (10 μM) and incubated under 5% CO_2_, at 37°C in a humidified atmosphere for 48 h. At the end of the treatment period, the cells were plated at a density of 5 × 10^3^ cells/well in the upper chamber of the Boyden chamber assembly and 10% FBS in the lower chamber was used as a chemoattractant. Separately, the cells in the upper chamber were treated with Doxorubicin (0.01–0.1 μM) alone or with the potential EGFR inhibitors (compounds—1c, 1e, 1g, 1h, 1j, and 1k (5 μM) based on their low IC50 values) or a commercial EGFR inhibitor, PD153035 (10 μM) and incubated for 4 h followed by fixation and staining as described previously.^28^, ^29^

### 2.7 Mammosphere forming efficiency

TNBC cell lines—*MDA‐MB‐231*, *MDA‐MB‐468*, or the sorted CD24^+^‐breast cancer cells, and CD24^−^/CD44^+^‐breast CSC populations from *MDA‐MB‐231* ^6^ were seeded at a 1 × 10^4^ cells/well density in mammosphere‐specific medium—Medium‐171 (Gibco, USA), supplemented with mammary epithelial growth supplement (MEGS) and B‐27 (Life technologies, Auckland, New Zealand) as described previously.^6^, ^26^ TNBC cells or the sorted breast cancer cells and CSCs were plated onto poly‐hydroxyethyl methacrylate (Sigma‐Aldrich, USA) coated 24‐well plates and subjected to treatment with DMSO (Vehicle), Doxorubicin (0.01 and 0.1 μM) alone or with the potential EGFR inhibitors (compounds—1c, 1e, 1g, 1h, 1j, and 1k (5 μM) based on their low IC50 values) or a commercial EGFR inhibitor, PD153035 (10 μM). The suspension cultures were incubated under 5% CO_2_, at 37°C in a humidified atmosphere for 7 days. At the end of the treatment period, the images of mammospheres were taken at 10× magnification using a microscope (Olympus IX71, Tokyo, Japan). The size of spheres was measured using ImageJ software (NIH, Bethesda, MD, USA).

### 2.8 Apoptosis assay

*MDA‐MB‐231* cells were seeded at a density of 3 × 10^4^ cells/well and cultured on a coverslip for 24 h at 37°C followed by 12 h serum starvation in 6‐well plates. The cells were treated with Doxorubicin (0.01 and 0.1 μM) in the absence or presence of compounds—1c, 1e, 1g, 1h, 1j, and 1k and incubated for 48 h. Post‐treatment, the cells were fixed using 4% Paraformaldehyde and stained with Hoechst 33342 dye (2 mg/mL; Invitrogen, USA) for 30 min. Apoptotic cells were identified by visualizing the fragmentation and condensation of nuclei using a confocal microscope (Olympus, USA).^30^

### 2.9 Gene expression studies

*MDA‐MB‐231* breast cancer cells plated at a density of 0.5 × 10^6^ cells/well onto 6‐well plates were treated with doxorubicin (0.01 and/or 0.1 μM) in the absence/presence of the selected potential EGFR inhibitors‐ 1c, 1e, 1g, 1h, 1j, and 1k (5 μM) for 24 h. To determine the level of EGFR expression the TNBC cells—*MDA‐MB‐231*, *MDA‐MB‐468*, and control *HEK293* cells were seeded at a density of 0.3 × 10^6^ cells/well onto 6‐well plates followed by 24 h of growth period separately. Briefly, the cells were incubated at 37°C with serum starvation followed by culturing as described above. Semi‐quantitative RT‐PCR was carried out with the isolated total RNA of *MDA‐MB‐231*, *MDA‐MB‐468*, and *HEK* cells.^28^, ^29^ Briefly, the template RNA (1 μg) was utilized for cDNA synthesis using a First‐strand cDNA synthesis kit (Thermo Scientific, USA) according to the manufacturer's protocol. Thermal cycler (Mastercycler® nexus, Eppendorf) was used for the amplification of the expression of apoptotic genes—Bax, Bcl2, Cytochrome‐C, and target gene EGFR with the help of Dream Taq DNA Polymerase PCR Master Mix (Thermo Scientific, USA) along with human gene‐specific forward and reverse primer sequences of the apoptotic genes and EGFR (Table ST1). The expression of the target genes was normalized to the Eukaryotic 18S rRNA.

Additionally, total RNA was isolated from tumor tissues and utilized for cDNA synthesis (Thermo Scientific, Massachusetts, USA). To determine the transcriptional regulation of expression of EMT genes in tumor tissues subjected to various treatment regimes, quantitative real‐time PCR (qRT‐PCR) analysis was performed as described previously^28^ using human gene‐specific forward and reverse primers of EMT genes. Amplification of Eukaryotic 18S rRNA served as an internal control and was utilized to calculate the fold change in expression.^29^

### 2.10 Immunoblot analysis

*MDA‐MB‐231* cells were subjected to the treatment of doxorubicin (0.01 μM) in the absence/presence of the selected potent compounds—1c, 1e, 1g, 1h, 1j, and 1k at 5 μM concentration or a standard EGFR inhibitor, PD153035 (10 μM) for 48 h. Separately, TNBC cells—*MDA‐MB‐231* were also treated with doxorubicin (0.01 and/or 0.1 μM) in the absence/presence of potent EGFR inhibitor/compound, **1e** at 5 μM concentration or a standard EGFR inhibitor, PD153035 (10 μM) for 48 h. Cells were then lysed to isolate the total protein and subjected to SDS‐PAGE, transferred onto a poly (vinylidene difluoride) membrane, probing with primary antibodies (Table ST2) against apoptotic markers—BAX, BCL‐2, cytochrome‐C, and β‐actin followed by incubation with horseradish peroxidase‐conjugated secondary antibodies (CST, Waltham, MA, USA) as described previously.^6^ Visualization was performed using G: Box (Syngene, Cambridge, UK).

### 2.11 Preclinical orthotopic xenograft murine tumor model

Animal studies have been carried out with the prior approval of the Institutional Animal Ethics Committee (IICT/IAEC/025/2023). *C57BL/6* female mice of age 6–8 weeks (*N* = 35) were initially immunosuppressed with cyclophosphamide (50 mg/kg of body weight, *i*.*p*) on alternate days for 2 weeks. For generating a preclinical orthotopic xenograft tumor model, 2 × 10^6^ GFP expressing sorted stable CD24^+^‐breast cancer cells and CD24^−^/CD44^+^‐breast CSCs were orthotopically xeno‐transplanted *subcutaneously* at the fourth mammary fat pad of immune‐suppressed mice (*n* = 5/group). Subsequently, *intratumoral* administration of Doxorubicin at a low dose (2.5 mg/kg of body weight), high dose (10 mg/kg of body weight), and/or combinatorial treatment of the potent EGFR inhibitor, compound **1e** (50 mg/kg of body weight) or the commercial EGFR inhibitor, PD153035 (50 mg/kg of body weight) with a low dose of doxorubicin was performed to the palpable tumors of breast CSCs at day‐3 post‐transplantation. Mice were observed to determine the tumor size (diameter) at regular intervals and calibrated using Vernier calipers every alternate day till day 14 while the tumor weight was evaluated post‐euthanization of the mice on day 14.^5^, ^31^

#### 2.11.1 Analysis of tumor tissues

##### Histological analysis

Euthanization of mice was performed on day 14 post‐transplantation and tumor tissues were collected for histological and immunohistochemical analysis. Histological differences in tumor tissues were evaluated using hematoxylin and eosin staining (H&E).^28^, ^30^

##### Immunohistochemical analysis

Immunohistochemical analysis was performed by immunostaining the tissue serial sections with primary antibodies against GFP, CD44, p‐EGFR, p‐JNK, and p‐p38 (CST, USA) followed by alexafluor‐488/555 conjugated secondary antibodies (R&D systems, USA) and mounting medium containing DAPI.^28^, ^29^ Images were captured using a confocal microscope (Olympus FV10i, Tokyo, Japan). Pearson's correlation coefficient was utilized to determine the co‐localization of GFP/CD44, GFP/p‐EGFR, GFP/p‐JNK, and GFP/p‐p38 (Table. ST3).

### 2.12 Statistical analysis

The data are represented as the mean ± SEM of the three independent experiments. Photomicrographs represent experiments performed in triplicates with reproducible results. ImageJ software was used for the quantitative analysis. Statistical analyses were performed to find out the differences between the treatment groups and their respective controls, using two‐way ANOVA followed by Tukey's analysis in PRISM, version 9, or student's paired *t* test. *p ≤ .05 was considered statistically significant.

## 3 RESULTS

### 3.1 Molecular docking scores of the inhibitors

**FIGURE 1:**
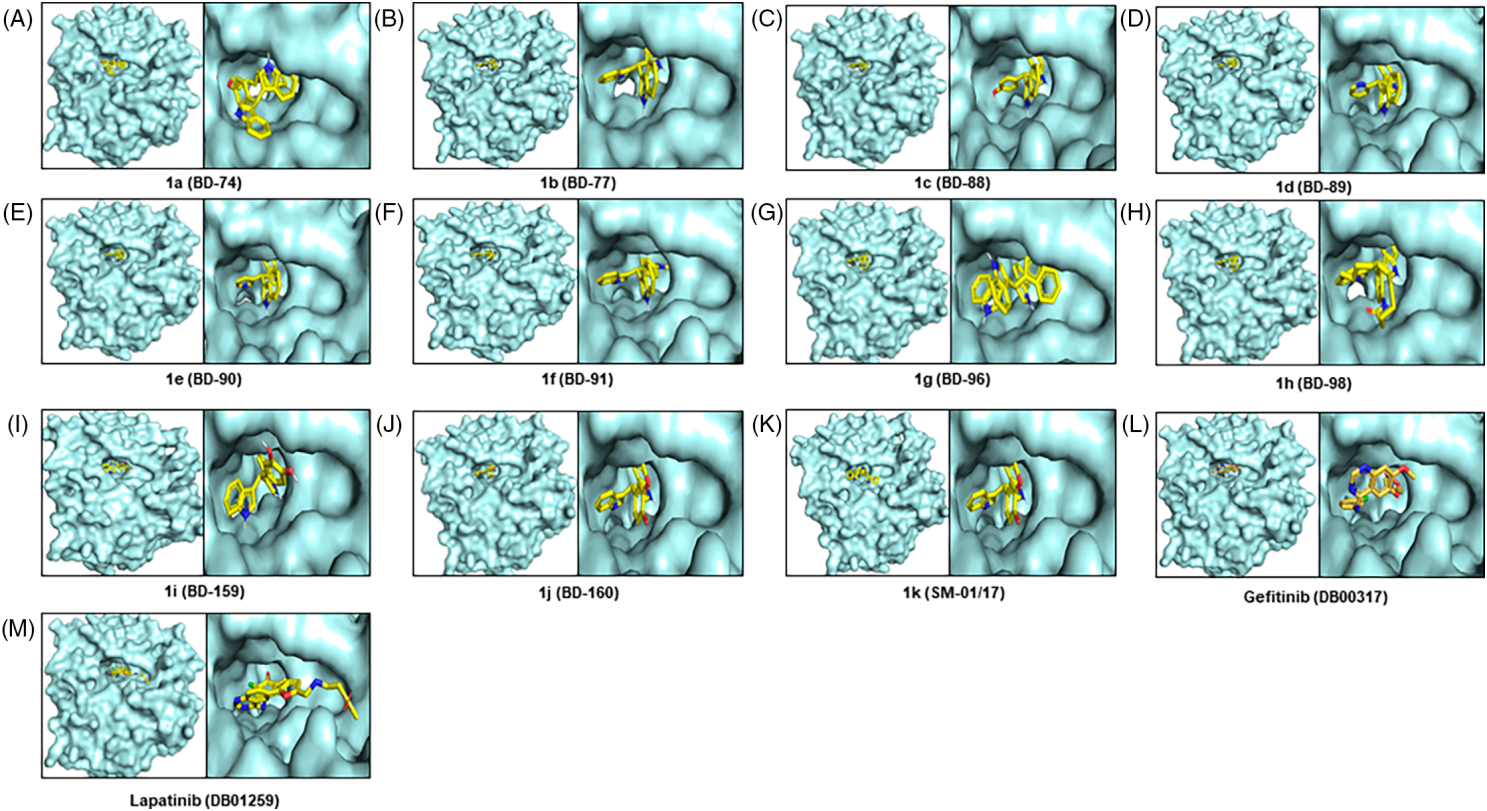
Molecular interaction between all the designed‐synthesized inhibitors of epidermal growth factor receptor (EGFR) protein and their binding positions on EGFR. The EGFR inhibitors are as follows: (A) 1a, (B) 1b, (C) 1c, (D) 1d, (E) 1e, (F) 1f, (G) 1g, (H) 1h, (I) 1i, (J) 1j, (K) 1k, (L) DB00317 (Gefitinib), and (M) DB01259 (Lapatinib).

The docking scores ranged from −8.5 to −12.7 for the compounds (potent EGFR inhibitors) as predicted by Autodock Vina (Table ST1). The docking scores of known EGFR inhibitors, Gefitinib and Lapatinib, were −7.9 and −8.4, respectively. Interestingly, these compounds showed a higher affinity for EGFR binding than the two known inhibitors. Molecular docking and molecular dynamics studies of an EGFR inhibitor revealed that the MET‐793, ASP‐800, and LEU‐844 amino acid residues were responsible for H‐bonding, while the PHE‐856 amino acid residue was involved in π‐π stacking interactions. MD simulation study also demonstrated that the amino acid residues MET‐793, ASP‐800, and LEU‐844 of EGFR are crucial residues for inhibiting EGFR effectively (Figure 1A–M). The 2‐D ligand interaction diagram demonstrated that the molecules 1c, 1i, and 1j interacted with Met‐793. Compounds 1a, 1b, 1c, 1d, 1e, 1f, 1g, 1h, 1j, and 1k interacted with other important amino acid residues Leu‐844. The only compound that interacted with Asp‐800 was 1i (Figure S1). A summary of interacting amino acid residues is displayed in Table ST3.

The IC50 values of best compounds 1k, **1e**, 1b, and known/commercial EGFR inhibitor PD153035 were 14.65, 18.64, 22.13, and 3.20^6^ determined against *MDA‐MB‐231* and their binding energy predicted by ADV were −10.4, −10.0, −11.5 kcal/mol, and −8.3 kcal/mol, respectively (Table 1). The binding free energy of compounds 1k, 1e, 1b, and PD153035 are corroborating with the experimental results. The colors of compounds 1k, **1e**, 1b, and PD153035 were lime green, magenta, cyan, and yellow, respectively. The binding poses of these compounds were observed to be similar to that of PD153035. The binding pose of one di‐indole portion of 1,4‐bis(di(1H‐indol‐3‐yl)methyl)benzene (1k) matched with **1e**, 1b, and PD153035. The other portion of the bi‐indole of **1e** is bound in different poses in the active site. The IC50 values of compounds 1c, 1g, 1k, and PD153035 were 24.98, 29.64, 20.36, and 15.4,^6^ respectively determined against *MDA‐MB‐468*. The binding energy of 1c, 1g, PD153035, and 1k were − 10.6, −10.0, −8.5, and −10.4 kcal/mol (Table 1). These binding energies also corroborate with the experimental IC50 values except for the known inhibitor PD153035. The binding poses of 1i, and PD153035 were very close and the hydroxyl group/methoxy groups were also in a similar direction. Therefore, the activities of these two compounds were much closer. The binding pose of all the inhibitors is shown in Figure S2A–D.

**TABLE 1:** Effect of designed‐synthesized small molecules as potential epidermal growth factor receptor inhibitors on proliferative potential of breast cancer and control cells.

S. no.	Name of the compounds	AD score (kcal/mol)	ADV score (kcal/mol)	IC_50_ (μM) in *MDA‐MB‐231* (mean ± SD)	IC_50_ (μM) in *MDA‐MB‐468* (mean ± SD)	IC_50_ (μM) in *HEK‐293* (mean ± SD)
1	1a	−7.83	−8.5	≥1000	108.64 ± 8.11	≥1000
2	1b	−10.38	−11.5	22.13 ± 1.96	90.80 ± 7.87	231.4 ± 15.9
3	1c	−9.12	−10.6	30.54 ± 2.93	24.98 ± 2.41	126.6 ± 16.8
4	1d	−9.49	−9.9	44.44 ± 2.11	47.63 ± 5.99	218.7 ± 11.6
5	1e	−9.43	−10.0	18.64 ± 1.84	35.98 ± 5.91	278.4 ± 13.5
6	1f	−9.65	−10.4	73.05 ± 1.13	129.76 ± 7.76	≥1000
7	1 g	−10.87	−10.3	≥1000	29.64 ± 6.04	≥1000
8	1 h	−9.88	−8.3	971.1 ± 151.4	98.90 ± 0.59	≥1000
9	1i	−9.92	−9.1	149 ± 9.17	129.6 ± 11.00	838.9 ± 26.6
10	1j	−9.13	−10.3	113.7 ± 1.25	68.38 ± 5.51	327.4 ± 20.7
11	1 k	−11.42	−10.4	14.65 ± 1.36	20.36 ± 2.41	235 ± 12.5

### 3.2 Cytotoxicity profile of the potential EGFR inhibitors in TNBC cells

These small molecules as potential EGFR inhibitors were evaluated for their cytotoxicity activities in TNBC cell lines—*MDA‐MB‐231* (Figure S3A), *MDA‐MB‐468* (Figure S3B), and non‐tumoral (control) cell line—*HEK293* (Figure S3C) by MTT assay, and the IC50 values have been enumerated in Table 1. To understand the mode of action of the compounds in these cell lines, we evaluated the expression levels of EGFR in the TNBC cell lines—*MDA‐MB‐231* and *MDA‐MB‐468* and non‐tumoral control cell line *HEK293*. Interestingly, we observed markedly high levels of EGFR expression in *MDA‐MB‐231*, and *MDA‐MB‐468* cells as compared with *HEK293* cells (Figure S4). This data supports the differential cytotoxic effect of these compounds (potential EGFR inhibitors) against the human cancerous and normal epithelial cells.

Further, a standard chemotherapeutic drug, Doxorubicin was utilized to elucidate the role of these selected compounds such as 1c, 1e, 1g, and 1h (potential EGFR inhibitors) in sensitization of TNBC cells toward chemotherapeutics. A differential increase in sensitization of the TNBC cells—*MDA‐MB‐231* and *MDA‐MB‐468* by these potential EGFR inhibitors (compounds) was observed with the combinatorial treatment of selected compounds at 5 μM concentration and doxorubicin at 0.01 μM concentration (Figure S5). Additionally, a remarkable increase in percent inhibition of the TNBC cells—*MDA‐MB‐231* and *MDA‐MB‐468* by the six potential EGFR inhibitors (compounds) was observed with the increasing doses of the selected compounds such as 1c, 1e, 1g, and 1h (1–10 μM) in combination with doxorubicin 0.01 μM as compared to the treatment of doxorubicin alone. A relatively higher percent inhibition of TNBC cells proliferation was observed at 5 and 10 μM concentration of these EGFR inhibitors (Table ST4). An insignificant difference in percent inhibition of TNBC proliferation between the at 5 and 10 μM concentration led us to utilize 5 μM concentration of these compounds for the combinatorial treatment of chemotherapeutics and the potential EGFR inhibitors in subsequent experiments. Two different kinds of molecules (doxorubicin—a chemotherapeutic drug, and compounds—potential EGFR inhibitors) were used for the combinatorial treatment. The inhibitors and Doxorubicin were used at non‐equimolar concentration (Doxorubicin −0.01 μM, below its IC50 values, 0.1 μM (*MDA‐MB‐231*) and 0.4 μM (*MDA‐MB‐468*), and the compounds (EGFR inhibitors) at 5 μM in *MDA‐MB‐231 and MDA‐MB‐468*) depending upon the observation as described above (Table ST4) that will potentially reduce any adverse reactions, therapeutically.

The breast cancer cells that were treated with the combination of chemotherapeutics and compounds such as 1c, 1e, 1g, and 1h showed a marked decrease in the IC50 values by greater than 10–100‐folds as compared to the cells treated with only doxorubicin in *MDA‐MB‐231* cells, indicating that these compounds increase the efficacy of doxorubicin (Table 2). Interestingly, the results also showed a 10‐fold decrease in the IC50 of Doxorubicin in the presence of the compounds 1c, 1e, 1g, 1h, 1j, and 1k as compared to Doxorubicin treatment alone in the *MDA‐MB‐468* cells, suggesting that these compounds increase the efficacy of doxorubicin (Table 2). In the present study, the inhibitors and Doxorubicin together showed a synergistic effect on the TNBCs that potentiated the efficacy of chemotherapeutics at a lower dose (0.01 μM).

**TABLE 2:** Treatment of triple‐negative breast cancer (TNBC) cells (*MDA‐MB‐231* and *MDA‐MB‐468*) with a chemotherapeutic drug in the presence/absence of potential epidermal growth factor receptor inhibitors.

S. no.	Groups	IC_50_ (μM) in *MDA‐MB‐231* (mean ± SD)	IC_50_ (μM) in *MDA‐MB‐468* (mean ± SD)
1	Doxorubicin	0.11 ± 0.01	0.40 ± 0.008
2	Doxorubicin + 1a	4.75 ± 1.47	0.84 ± 0.01
3	Doxorubicin + 1b	0.106 ± 0.004	1.35 ± 0.08
4	Doxorubicin + 1c	0.001 ± 0.0002	0.007 ± 0.001
5	Doxorubicin + 1d	0.26 ± 0.03	0.12 ± 0.003
6.	Doxorubicin + 1e	0.03 ± 0.04	0.01 ± 0.0005
7	Doxorubicin + 1f	0.10 ± 0.01	0.18 ± 0.005
8.	Doxorubicin + 1g	0.0006 ± 0.0001	0.01 ± 0.0002
9	Doxorubicin + 1h	0.03 ± 0.003	0.01 ± 0.0004
10	Doxorubicin + 1i	8.89 ± 1.69	6.92 ± 1.33
11	Doxorubicin + 1j	0.25 ± 0.009	0.05 ± 0.001
12	Doxorubicin + 1k	0.24 ± 0.02	0.04 ± 0.002

### 3.3 EGFR inhibition modulates migratory capacity in TNBC cells

Both the TNBC cell lines *MDA‐MB‐231* and *MDA‐MB‐468* depicted a significant decrease in the migratory potential in the presence of combinatorial treatment of low dose of Doxorubicin (0.01 μM) with the EGFR inhibitors (compounds—1c, **1e**, and 1g at 5 μM) or known EGFR inhibitor, PD153035 (10 μM) as compared to the positive control (10% FBS) and Doxorubicin treatment alone (0.01 μM) groups (Figure S6A and S6B). This suggests a key role of the compounds—1c, **1e**, and 1g in abrogation of the migratory potential in aggressive TNBCs.

### 3.4 EGFR inhibition modulates mammosphere formation ability in TNBC cells

The in‐vitro tumorigenesis property of cancer cells as assessed by the mammosphere‐forming capability, can be associated with the in‐vivo tumor‐forming efficiency. Therefore, to further corroborate the cytotoxicity profile of the compounds, we assessed the mammosphere‐forming capability of breast cancer cells treated with doxorubicin in the absence or presence of the selected compounds—1c, 1e, 1g, 1h, 1j, and 1k as potential EGFR inhibitors, based on their low IC50 values in *MDA‐MB‐231* and *MDA‐MB‐468* breast cancer cells.

The quantitative analysis of mammospheres in *MDA‐MB‐231* cancer cells suggested a significant lowering in sphere‐formation efficiency of doxorubicin in the presence of the compounds as compared to the cells treated with doxorubicin alone (Figure S7A). There was a marked decrease in sphere‐forming abilities by doxorubicin in the presence of these selected potential EGFR inhibitors in *MDA‐MB‐468* (Figure S7B). This observation indicates that the selected compounds may play a crucial role in potentiating the anti‐tumorigenic effect of doxorubicin.

### 3.5 EGFR inhibitors enhance cellular apoptosis by modulating the expression of apoptotic genes and proteins in aggressive TNBCs

**FIGURE 2:**
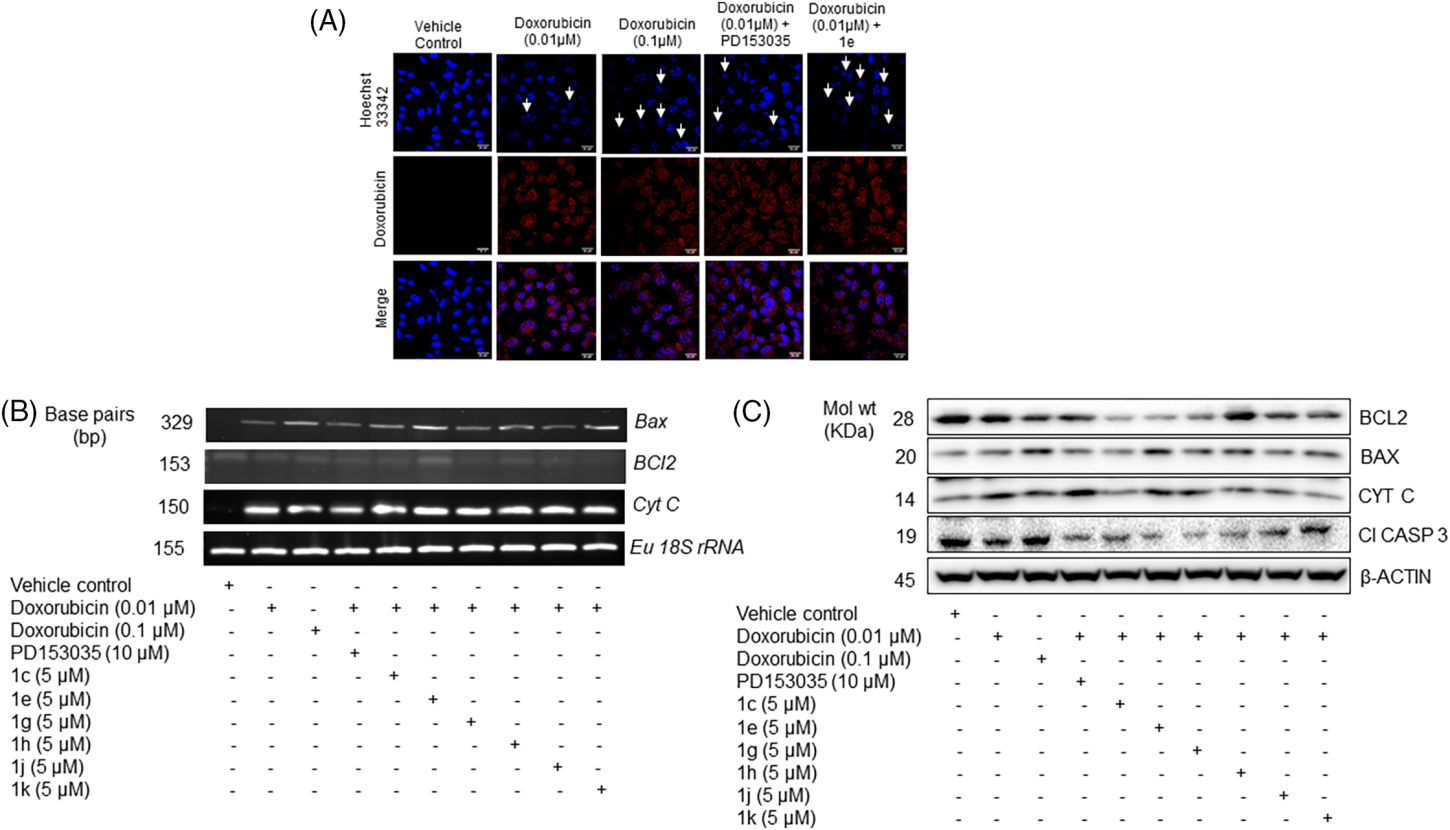
Detection of apoptosis induced by the combinatorial treatment of doxorubicin and selected compounds/epidermal growth factor receptor (EGFR) inhibitors. (A) Representative images depicting the apoptosis of *MDA‐MB‐231* cells stained with Hoechst 33342. Data are represented as the results of three independent experiments (*n* = 3). (B) Modulation of aggressive triple‐negative breast cancer (TNBC) apoptotic genes by EGFR inhibitors. (C) Modulation of aggressive TNBC apoptotic proteins by EGFR inhibitors. Data are represented as the results of three independent experiments (*n* = 3).

*MDA‐MB‐231* cells treated with doxorubicin in the absence/presence of compounds 1c, 1e, 1g, 1h, 1j, and 1k were subjected to staining with Hoechst 33342 that exhibited chromatin condensation and nuclear fragmentation (Figure 2A and Figure S8). Combinatorial treatment of compound **1e** markedly increased the doxorubicin‐induced nuclear fragmentation as compared with the other treatment groups, indicating increased apoptosis (Figure 2A). Next, we evaluated expression levels of apoptotic genes using human‐specific gene primers (Table ST1). Corroborating with the cellular apoptosis observations, a marked increase was observed in the expression of molecular pro‐apoptotic genes—Bax and Cytochrome‐C in *MDA‐MB‐231* cells with the combinatorial treatments of chemotherapeutic drug—doxorubicin and the potent EGFR inhibitors/compounds—1c, 1e, 1g, 1h, 1j, and 1k or PD153035 (Figure 2B) suggesting that EGFR inhibition potentiates doxorubicin‐mediated cell death.

The pro‐apoptotic Bax and the anti‐apoptotic BCL‐2 proteins regulate cellular apoptosis. *MDA‐MB‐231* cells were treated with doxorubicin (0.01 μM) in the absence/presence of the potential EGFR inhibitors/compounds—1c, 1e, 1g, 1h, 1j, and 1k. Compound **1e** showed a marked decrease in the BCL2 levels in *MDA‐MB‐231* cells, (Figure 2C, S9A, S10) elevation in BAX (Figure 2C, S9B, S10), and BAX/BCL2 ratio (Figure S9C), suggesting its ability to elevate the therapeutic response of doxorubicin in *MDA‐MB‐231* cells. Similarly, a differential effect on the expression of Cytochrome‐C was observed with the treatment of doxorubicin (0.01 μM) in the presence of these compounds (Figure 2C, S9D, S10). Compound **1e** induced the doxorubicin‐induced expression of Cyt‐C at comparatively higher levels than the others suggesting the activation of the intrinsic apoptotic pathway (Figure 2C). Although the compounds 1g and 1h also showed a comparative efficacy in reducing the physiological effects in TNBCs, the higher expression of BAX gene and protein levels, decreased expression of BCL‐2, along with the BAX/BCL2 quantitative ratio (Figure S9A–C) in the presence of compound **1e** among all the six evaluated EGFR inhibitors (compounds) led us to evaluate the in‐vitro and in‐vivo efficacy of this candidate **1e** on the sorted populations of breast CSCs from TNBCs.

### 3.6 Antiproliferative effect of compound 1e against sorted breast cancer cell and CSC populations

**FIGURE 3:**
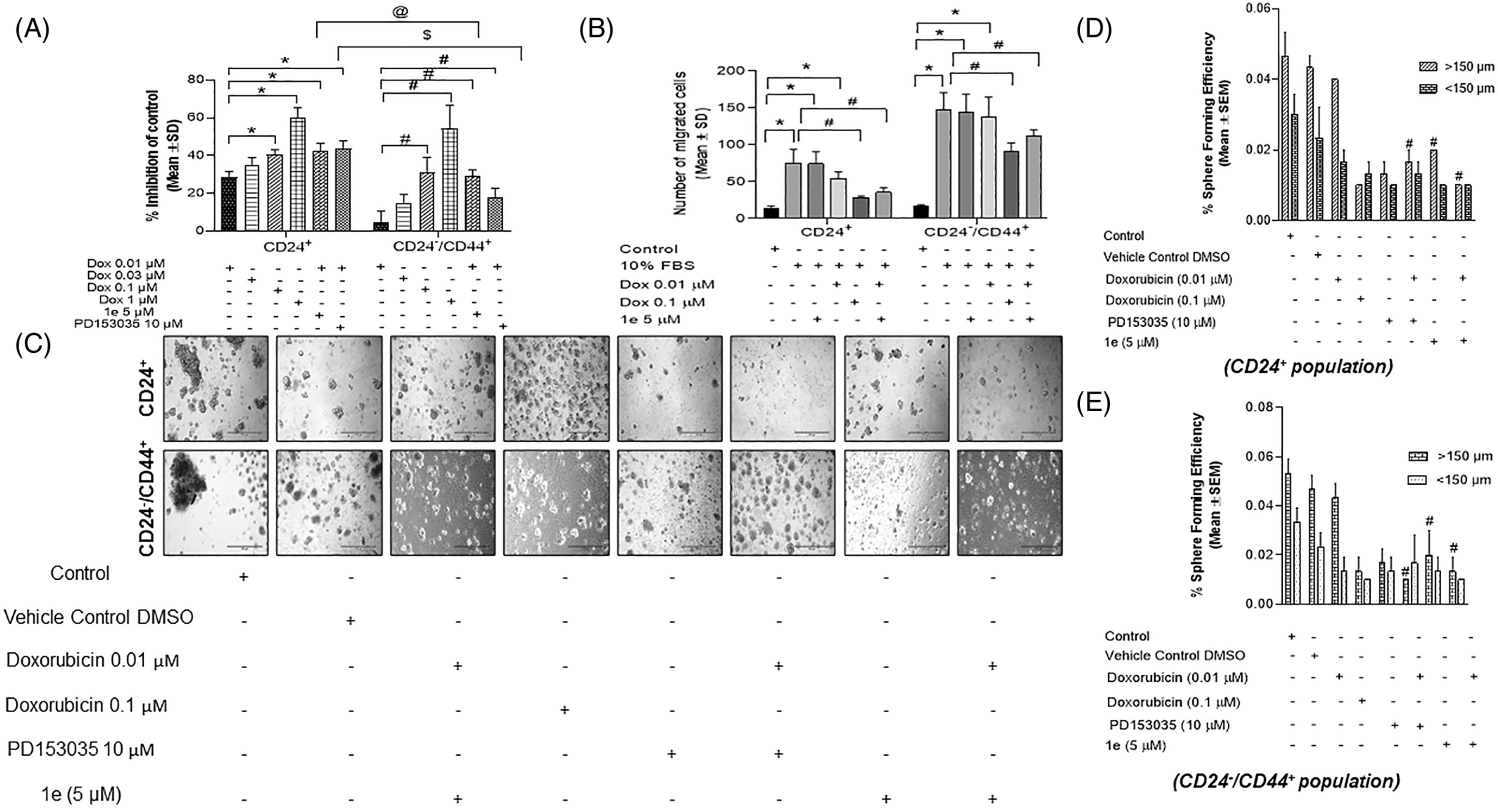
Combinatorial treatment‐mediated regulation of cell proliferation, migration, and in‐vitro tumorigenesis in breast cancer stem cells (CSCs) isolated from TNBCs. (A) Proliferative potential of CD24^−^/CD44^+^ breast CSCs and CD24^+^ breast cancer cells sorted from TNBC *MDA‐MB‐231* cells in the presence of combinatorial treatment of Doxorubicin with 1e and PD153035 (*n* = 6). Data represents the percent inhibition as mean ± SD of three independent experiments. *p* ≤ .05 as compared to *Doxorubicin (0.01 μM) group. (B) Graph depicting the migratory potential of CD24^−^/CD44^+^‐breast CSCs and CD24^+^‐breast cancer cells in the presence of combinatorial treatment of Doxorubicin with 1e and PD153035 (*n* = 4). Data represents the number of cells migrated as mean ± SD of three independent experiments. *p* ≤ .05 as compared to *control and ^#^10% FBS group. (C) epidermal growth factor receptor (EGFR) inhibition modulates the mammosphere formation efficiency in both sorted breast cancer cells and CSCs. Representative photomicrographs of mammospheres depicting the differences in sphere‐forming abilities with the treatment of a chemotherapeutic drug in the presence/absence of potential EGFR inhibitor 1e in CD24^+^ and CD24^−^/CD44^+^ sorted populations isolated from MDA‐MB‐231. Quantification of the mammosphere images of both sorted (D) CD24^+^ and (E) CD24^−^/CD44^+^ population. Data represents the percent sphere forming efficiency as mean ± SEM of three independent experiments. *p* ≤ .05 as compared to ^#^Doxorubicin (0.01 μM) group.

The percent inhibition of cell proliferation of the sorted CD24^+^‐breast cancer cells and CD24^−^/CD44^+^‐breast CSC populations in the presence of combinatorial treatment of doxorubicin (0.01 μM) and **1e** (5 μM) was observed to be increased by 6.1‐folds in CD24^−^/CD44^+^ breast CSC population and 1.8‐folds in CD24^+^ cancer cells as compared with doxorubicin (0.01 μM) treatment alone (Figure 3A). The compound, **1e** depicted an increased percent inhibition as compared to the standard EGFR inhibitor, PD153035 (10 μM) along with the combinatorial treatment of doxorubicin (Figure 3A). This suggests a potential role of the compound **1e** in inhibiting the cell proliferation of breast CSCs‐mediated tumorigenesis.

### 3.7 Anti‐migratory effect of compound 1e against sorted breast cancer cell and CSC populations

The migratory potential of the sorted CD24^+^‐breast cancer cells and CD24^−^/CD44^+^‐breast CSC populations in the presence of combinatorial treatment of Doxorubicin low dose (0.01 μM) and compound **1e** (5 μM) was observed to be significantly reduced as compared to the positive control group (10% FBS) (Figure 3B). The chemotactic potential of both sorted breast cancer cells and CSCs treated with 10% FBS was higher as compared to respective control. However, the migratory potential of breast CSCs was higher as compared with breast cancer cells (Figure 3B). Interestingly, the EGFR inhibitor **1e** could significantly reduce the migratory potential of these sorted populations of breast cancer cells and CSCs treated with doxorubicin at low concentration (0.01 μM) that was comparable with high dose of Doxorubicin (0.1 μM) alone, which indicates the anti‐migratory potential of identified EGFR inhibitor **1e** in the presence of chemotherapeutics.

### 3.8 Inhibition of mammosphere formation efficiency of sorted breast cancer cell and CSC populations by compound 1e

The evaluation of in‐vitro tumorigenesis properties in sorted populations of CD24^+^‐breast cancer cells (Figure 3C,D) and CD24^−^/CD44^+^‐breast CSCs (Figure 3C,E) of *MDA‐MB‐231* depicted a significant decrease in the mammosphere forming efficiency in the presence of doxorubicin (0.01 μM) with the EGFR inhibitor/compound **1e** that was comparable to the commercial EGFR inhibitor (PD153035), which in turn was comparable to the doxorubicin alone at a high dose (0.1 μM) (Figure 3D,E). This indicates that compound **1e** plays an efficacious role in potentiating the doxorubicin‐mediated decrease in sphere‐forming capabilities of both the sorted populations.

### 3.9 EGFR inhibition therapeutically mitigates orthotopic xenograft of breast CSC‐induced tumorigenesis

**FIGURE 4:**
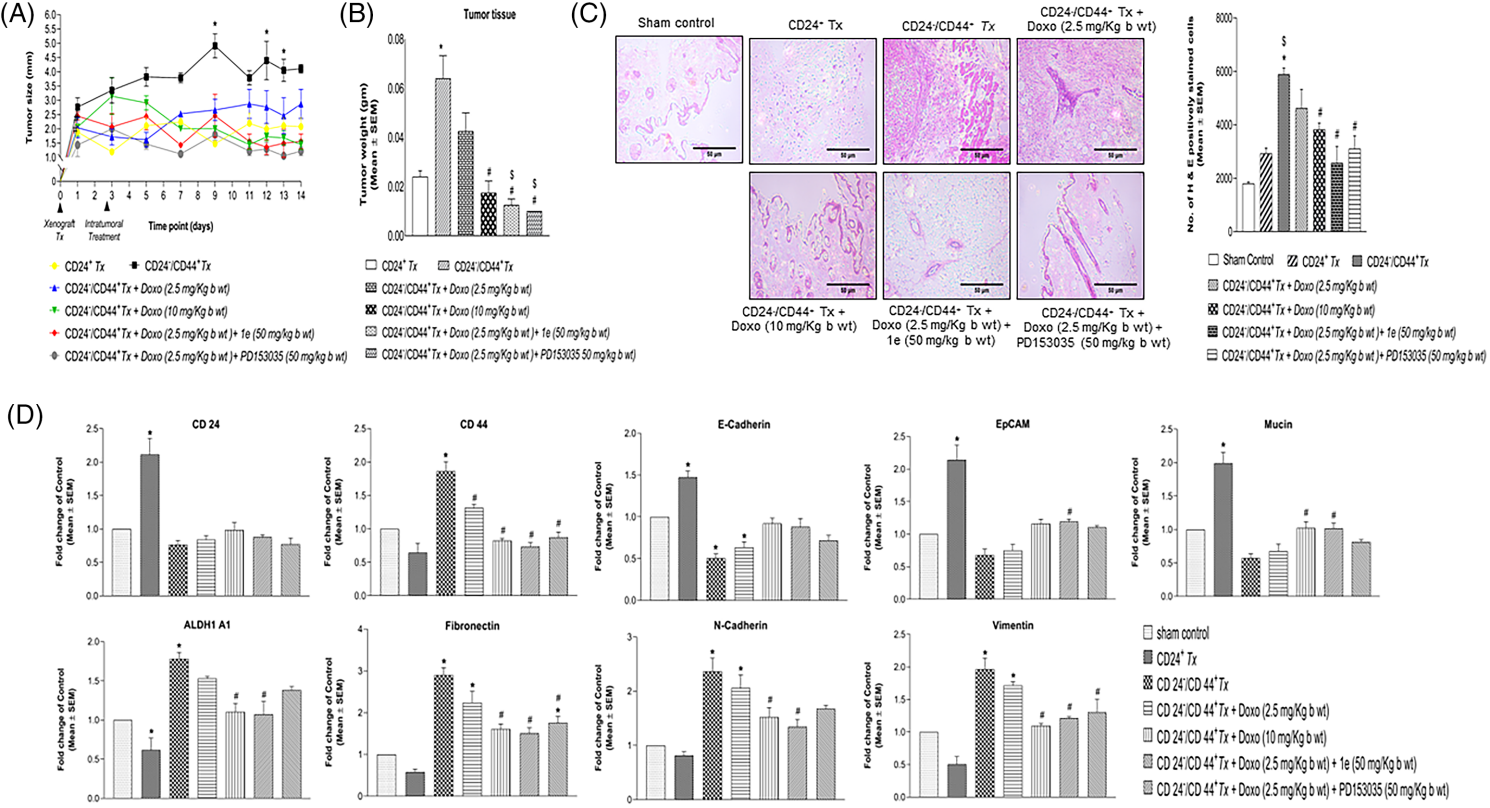
Inhibiting epidermal growth factor receptor therapeutically decreases the tumor growth in orthotopic xenograft in‐vivo model. Analysis of the (A) tumor size and (B) tumor weight in mice groups transplanted with breast cancer stem cells (CSCs) and breast cancer cells. (C) Representative images of hematoxylin and eosin‐stained tumor tissues (left panel) and quantification of images (right panel) depicted a marked increased number of H&E‐positive cells in tumor tissues of mice xenotransplanted with CD24^−^/CD44^+^‐breast CSCs as compared to the sham control as well as tumor tissues of mice transplanted with CD24^+^‐breast cancer cells (*p* < .05, as compared with *sham control, ^$^CD24^+^ *Tx*; ^#^CD24^−^/CD44^+^ *Tx*‐treated with Doxo (2.5 mg/kg bw). (D) Expression of human CSC‐specific genes in tumor tissues excised from *C57BL/6J* mice. Graphs depicting qRT‐PCR analysis of human *CD44*, *ALDH1A1*, *Fibronectin*, *N‐Cadherin*, *Vimentin*, *CD24*, *E‐Cadherin*, *EpCAM*, and *Mucin* gene expression in the tumor tissues of mice. *p < .05 compared to *sham control and ^#^CD24^−^/CD44^+^ *Tx*‐untreated group.

We developed an orthotopic xenograft tumor model in *C57BL/6* mice (*n* = 5) to understand the role of EGFR in tumorigenesis. 100% tumor incidence was observed in the mice transplanted with CD24^+^‐breast cancer cells and CD24^−^/CD44^+^‐breast CSCs. Mice transplanted with CD24^−^/CD44^+^‐breast CSCs exhibited a significant increase in tumor size (Figure 4A) and weight (Figure 4B) as compared with that of CD24^+^‐breast cancer cells, suggesting the high tumorigenic potential of breast CSCs as compared to cancer cells. Mice group treated with a high dose of doxorubicin (10 mg/kg of body weight) exhibited significantly lower tumor size and weight as compared with the untreated breast CSCs transplanted group. Furthermore, the *intratumoral* treatment of doxorubicin at a low dose (2.5 mg/kg of body weight) along with either the potent EGFR inhibitor/compound **1e** or standard EGFR inhibitor, PD153035 in mice xenotransplanted with CD24^−^/CD44^+^‐breast CSCs significantly decreased the tumor size as compared with a low dose of doxorubicin alone that was comparable to the high dose of doxorubicin treated group suggesting the enhanced therapeutic potential of EGFR inhibitor in reducing doxorubicin‐mediated breast tumor growth.

Histopathological analysis of mice tumor sections revealed a higher accumulation of necrotic debris correlating with the higher tumorigenic potential of the CD24^−^/CD44^+^‐breast CSCs as compared with CD24^+^‐breast cancer cells xenotransplanted groups (Figure 4C), which was quantified using Image J software (Figure 4C, right panel). Tissue sections of the untreated group showed normal morphology of mammary gland fat pads composed of white adipose tissue. This tumorigenic potential of CD24^−^/CD44^+^‐breast CSCs was perturbed with the *intratumoral* administration of a high dose (10 mg/kg body weight) but not at a low dose (2.5 mg/kg body weight) of doxorubicin (Figure 4C right panel). Further, in mice treated with a combination of a low dose of doxorubicin with the EGFR inhibitor/compound **1e**, and/or standard EGFR inhibitor, PD153035, a significant decrease in tumorigenicity was observed, thus suggesting the role of EGFR inhibition in potentiating doxorubicin‐mediated decreased tumorigenesis of breast CSCs. Tumors excised from the CD24^−^/CD44^+^‐breast CSCs xenotransplanted group treated with a low dose of doxorubicin alone depicted the presence of neoplastic epithelial cells that formed nodules in the dermal region and invaded to the adjacent subcutaneous region (Figure 4C). In the CD24^−^/CD44^+^‐breast CSCs xenotransplanted group treated with a high dose of doxorubicin, the tumor mass in the subcutaneous region was completely necrosed and replaced with fibrous tissue (Figure 4C). However, in CD24^−^/CD44^+^‐breast CSCs xenotransplanted group treated with a low dose of doxorubicin and the potent EGFR inhibitor/compound **1e**, normal morphology of mammary gland with subcutaneous fat was observed with no tumor mass (Figure 4C). In contrast, mice groups xenotransplanted with CD24^−^/CD44^+^‐breast CSCs and treated with a low dose of doxorubicin along with the standard EGFR inhibitor, PD153035 displayed neoplastic mass in the dermal region which was completely necrosed and replaced with fibrous tissue. These data suggest that the combinatorial treatment of a chemotherapeutic drug, doxorubicin at a low dose and the potent EGFR inhibitor/compound **1e** effectively regressed the tumor growth by inhibiting the tumorigenic potential of the breast CSCs.

### 3.10 EGFR inhibition perturbs the aggressive mesenchymal phenotype in breast CSCs

Next, we assessed the expression levels of breast CSC‐specific genes in the tumor tissue of *C57BL/6* mice using human‐specific primers (Table ST2) with no cross‐reactivity. A significant increase in human *CD44* mRNA expression levels was observed in tumor tissues of mice xenotransplanted with CD24^−^/CD44^+^‐breast CSCs as compared to sham control (Figure 4D). The expression level of *CD44* was observed to be significantly decreased in the mice group treated with doxorubicin low dose along with the potent EGFR inhibitor/compound **1e**, and/or the standard EGFR inhibitor, PD153035 as compared with the mice group treated with a low dose of doxorubicin, alone. The decrease in human *CD44* expression in the mice tumor tissues in the low dose of doxorubicin and compound **1e** or PD153035‐treated group was comparable to that of the high dose of the doxorubicin‐treated group (Figure 4D). Similarly, in mice groups xenotransplanted with CD24^−^/CD44^+^‐breast CSCs, the combinatorial treatment of potent EGFR inhibitor/compound **1e** and a low dose of doxorubicin significantly decreased the expression of human mesenchymal genes such as *ALDH1A1*, *Fibronectin*, *N‐Cadherin*, and *Vimentin* that was comparable to high dose of doxorubicin alone‐treated group suggesting that EGFR inhibition potentiates the therapeutic effect of doxorubicin. In contrast, a significant increase in the expression levels of human *CD24*, as well as the epithelial genes such as *E‐Cadherin*, *EpCAM*, *and Mucin*, was observed in tumor tissues of mice xenotransplanted with CD24^+^‐breast cancer cells (Figure 4D). These data suggest that EGFR inhibition effectively potentiates the anti‐tumorigenic effect of doxorubicin by inhibiting the mesenchymal characteristics of these aggressive phenotype in the breast CSCs.

**FIGURE 5:**
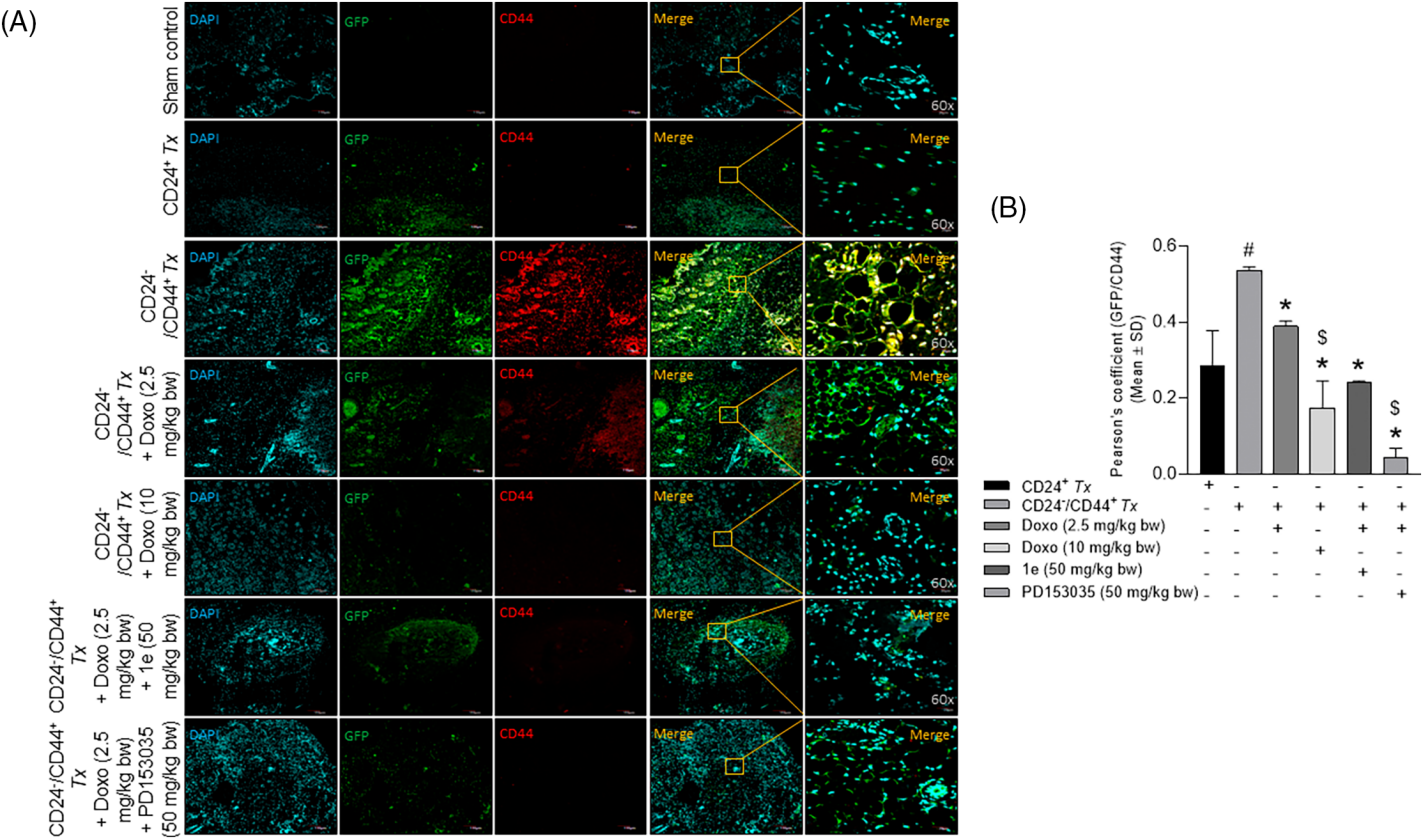
Epidermal growth factor receptor inhibition potentiates the doxorubicin‐mediated decreased tumorigenesis of xenotransplanted breast CSCs in the tumor tissues. (A) Representative confocal microscopy images of the tumor tissue section co‐immunostained with GFP/CD44 showing a significant increase in the (B) Pearson's correlation coefficient in CD24^−^/CD44^+^‐breast CSCs xenotransplanted group as compared to CD24^+^‐breast cancer cells xenotransplanted group, (Doxo‐Doxorubicin, *p* < .05, as compared with ^#^CD24^+^ *Tx*; *CD24^−^/CD44^+^ *Tx‐*untreated; ^$^CD24^−^/CD44^+^ *Tx*‐treated with Doxo (2.5 mg/kg body wt).

**FIGURE 6:**
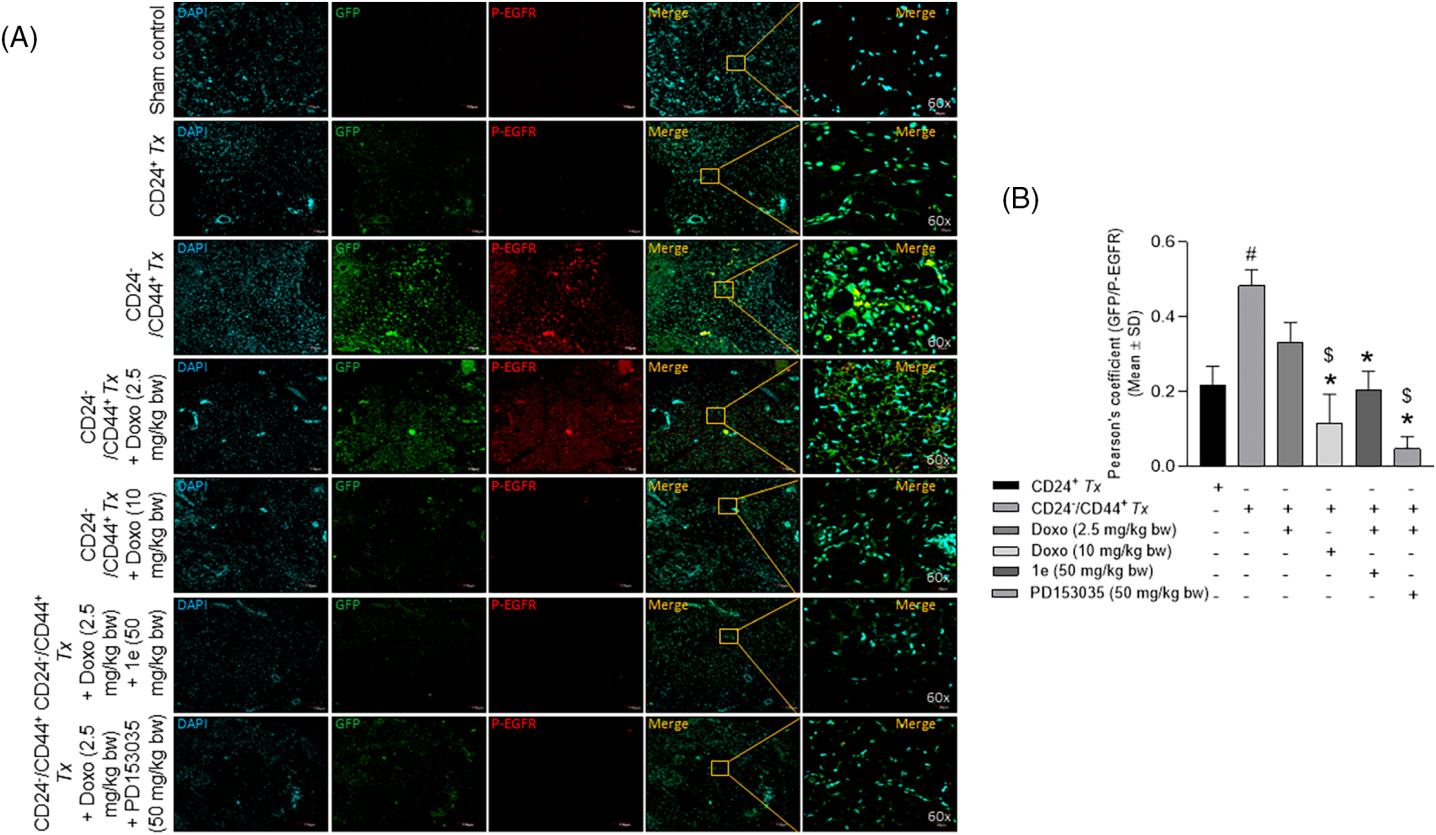
Activation of epidermal growth factor receptor (p‐EGFR) in the GFP‐expressing xenotransplanted cells within the tumor tissues. (A) Representative confocal microscopy images of the tumor tissue sections co‐immunostained with GFP/p‐EGFR showing a significant increase in the activation of EGFR (phosphorylated EGFR), evident from the (B) Pearson's correlation coefficient in CD24^−^/CD44^+^‐breast CSCs xenotransplanted group as compared to CD24^+^‐breast cancer cells xenotransplanted group (Doxo‐Doxorubicin, *p* < .05, as compared with ^#^CD24^+^ *Tx*; *CD24^−^/CD44^+^ *Tx‐*untreated; ^$^CD24^−^/CD44^+^ *Tx*‐treated with Doxo (2.5 mg/kg body wt).

**FIGURE 7:**
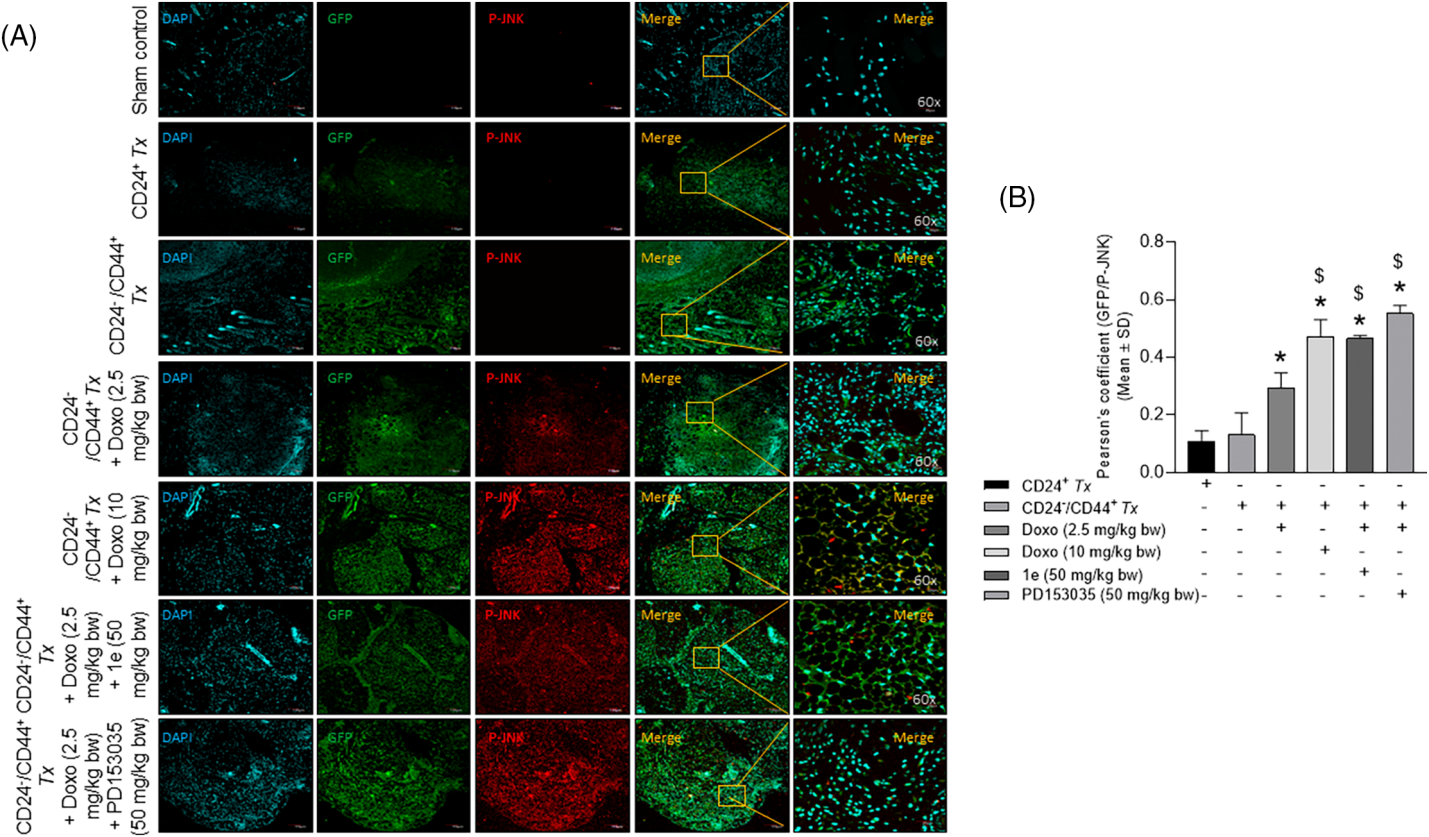
Epidermal growth factor receptor inhibition‐mediated doxorubicin‐induced apoptotic downstream signaling of JNK in the tumor tissues. (A) Representative confocal microscopy images of tumor tissue sections co‐immunostained with GFP/p‐JNK showing a significant increase in the activation of JNK (phosphorylated JNK), evident from the (B) Pearson's correlation coefficient in CD24^−^/CD44^+^‐breast CSCs xenotransplanted groups treated with doxorubicin at low dose in combination with compound 1e, and/or PD153035 (Doxo‐Doxorubicin, *p* < .05, as compared with *CD24^+^ *Tx*; ^$^CD24^−^/CD44^+^ *Tx*‐treated with Doxo (2.5 mg/kg body wt).

**FIGURE 8:**
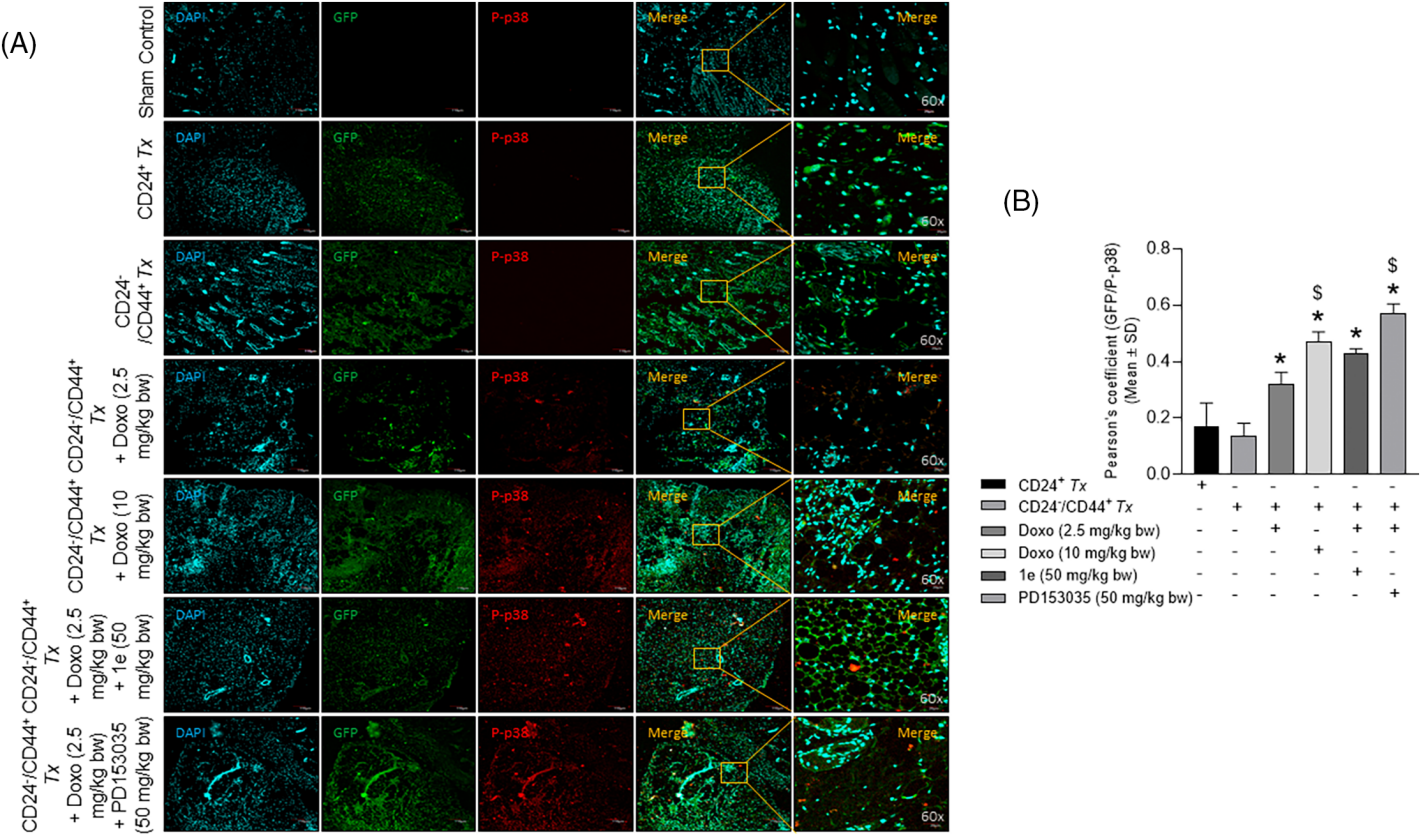
Epidermal growth factor receptor inhibition‐potentiated doxorubicin‐induced apoptotic downstream signaling of P38 in the tumor tissues. (A) Representative confocal microscopy images of tumor tissue sections co‐immunostained with GFP/p‐P38 showing a significant increase in the activation of P38 (phosphorylated P38), evident from the (B) Pearson's correlation coefficient in CD24^−^/CD44^+^‐breast CSCs xenotransplanted groups treated with doxorubicin at low dose in combination with compound 1e, and/or PD153035. (Doxo‐Doxorubicin, *p* < .05, as compared with *CD24^+^ *Tx*; ^$^CD24^−^/CD44^+^ *Tx*‐treated with Doxo (2.5 mg/kg body wt).

Immunohistochemical analysis of the tumor tissue sections with antibodies (Table ST3) revealed a significant increase in the colocalization of GFP and CD44 in the CD24^−^/CD44^+^‐breast CSC as compared with the CD24^+^‐breast cancer cell xenotransplanted groups (Figure 5A). A significant decrease in Pearson's correlation coefficient (Figure 5B) was observed in a dose‐dependent manner in the tumor tissue of CD24^−^/CD44^+^ xenotransplanted groups treated with low and high doses of doxorubicin as compared to the untreated groups (Figure 5A,B right panel). The combinatorial treatment of a low dose of doxorubicin and compound **1e** or PD153035 was comparable to the high dose of doxorubicin treated group (Figure 5A,B). Similarly, a significant increase in the colocalization of GFP and p‐EGFR (activated EGFR) was observed in the CD24^−^/CD44^+^‐breast CSC as compared with the CD24^+^‐breast cancer cell xenotransplanted groups (Figure 6A). Further, the colocalization of GFP/p‐EGFR decreased significantly in the doxorubicin at a low dose‐treated group in the presence of the potent EGFR inhibitor/compound **1e**, and/or the standard EGFR inhibitor, PD153035 that was comparable with the high dose of doxorubicin‐treated group (Figure 6B). Additionally, co‐immunostaining of GFP with the apoptotic signaling mediators p‐JNK and p‐p38 revealed a dose‐dependent significant increase in the colocalization of GFP/p‐JNK (Figure 7A,B) and GFP/p‐p38 (Figure 8A,B) in the CD24^−^/CD44^+^‐breast CSC xeno‐transplanted group treated at a low and high dose of doxorubicin as compared to the untreated group. Interestingly, increased activation of apoptotic signaling mediators with the combinatorial treatment of a low dose of doxorubicin with potent EGFR inhibitor/compound **1e**, and/or standard EGFR inhibitor, PD153035 was comparable with the high dose of doxorubicin alone (Figure 7A,B, 8A,B). These results further confirm the efficacy of compound **1e** as a potent EGFR inhibitor with tumor‐inhibiting properties.

## 4 DISCUSSION

Breast cancers are categorized based on their molecular profiling and their aggressive characteristics. As compared to the Her2‐positive breast cancer cell lines (*MDA‐MB‐453*, *SK‐BR‐3*), TNBCs (*MDA‐MB‐231*, *MDA‐MB‐468*), are the most aggressive ones due to the absence of targeted therapies in contrast to the luminal breast cancer cell lines (*MCF‐7*, *BT‐474*).^2^, ^32^ Response of TNBC patients toward conventional chemotherapeutic drugs like doxorubicin, tamoxifen, and so forth is very poor because of breast cancer recurrence.^33^, ^34^ However, breast cancer metastasis is considered a primary cause of the relapse of TNBC patients, due to the migration of breast CSCs, generated through Epithelial to Mesenchymal Transition (EMT) of breast cancer cells. This metastatic nature of breast CSCs ultimately reduces the overall disease‐free survival of breast cancer patients.^35^ The upregulation of EGFR is one of the known prognostic factors associated with the reduced survival of TNBC patients.^36^ There is evidence for the higher rate of metastasis in TNBC patients, which also eventually correlates with decreased survival of the patients compared to other breast cancer subtypes. Recent advancement in drug discovery has led to the development of several therapeutic approaches for the treatment of breast cancer such as monoclonal antibodies (cetuximab, Panitumumab), antisense oligonucleotides (Phosphorothioate oligodeoxynucleotides), and antibody‐based immuno‐conjugates (40H3‐Deruxtecan, IgG1‐Tesirine, IMGN289) apart from the EGFR‐TKIs (erlotinib and gefitinib), which act via down‐regulating the various signaling pathways.^37^, ^38^, ^39^, ^40^ The efficiency of EGFR‐TKIs used in targeted cancer treatment regimens is often restricted to the emergence of acquired resistance targeting drug‐resistant mutants of EGFR.^10^, ^11^, ^12^ Currently, the available EGFR‐TKIs generally target the ATP binding site of the EGFR kinase domain, necessitating the need for therapeutic agents with alternative mechanisms of action for overcoming resistance to EGFR mutants.^41^ Caporuscio et al. have shown the design and development of small affinity molecules by a cost‐effective, high throughput docking method for identifying allosteric interaction sites of EGFR.^42^ To date, several clinical trials have been performed to assess the efficiency of TKIs in breast cancer and the results are ineffective owing to the monotherapy treatment regime.^43^ The combinatorial treatment with pharmacological inhibitors of EGFR such as gefitinib, erlotinib, and PI3K/AKT pathway has exhibited a synergistic anti‐tumorigenic potential in treating basal subtypes of TNBCs, resulting in reduced metastasis of breast cancer cells.^44^ This observation correlates with our findings of combinatorial treatment with potent EGFR inhibitor **1e** along with chemotherapeutics effectively mitigated the cell proliferation and migration of sorted breast CSCs from aggressive TNBCs. GALNT8‐mediated O‐Gal N Acylation suppresses the EGFR signaling pathway and metastatic potential in breast cancer cells.^45^ Thus, a combinatorial treatment strategy of chemotherapeutics along with small molecule EGFR inhibitors might be a therapeutic way forward in the treatment of TNBC patients by inhibiting EGFR‐mediated tumorigenesis.

To identify efficacious small molecule inhibitors against EGFR with reduced side effects, our study has identified potent EGFR inhibitors using molecular docking studies. Our observation is in alignment with the prior reports of combinatorial treatment of Doxorubicin with a potential EGFR inhibitor was found to be effective in inhibiting the proliferation of TNBC cells.^46^ The aggressive properties of the breast cancer cells are evaluated using an in‐vitro assay system, where the cancer cells are cultured in a suspension medium, known as ‘mammospheres’ which mimic in‐vivo tumors.^47^ Our data suggest that the combinatorial treatment approach with the designed EGFR inhibitors sensitizes the breast CSCs toward chemotherapeutics, which corroborates well with the literature.^48^ The pro‐apoptotic proteins BAX (BCL‐2‐associated X protein), cytochrome‐C, and the anti‐apoptotic protein BCL‐2 (B‐cell lymphoma 2) play an important role in the process of apoptosis.^49^ BAX and BCL‐2 expression are regulated through the *p53‐*induced apoptosis pathway. Generally, apoptosis leads to a highly complex cascade of cellular events characterized by chromatin condensation, and DNA fragmentation.^50^ Corroborating with the literature, our study also demonstrated a plausible apoptotic effect on the TNBC cells by the potent EGFR inhibitor, **1e** in the presence of chemotherapeutics. It is noteworthy that in our preclinical orthotopic model, grafted/xenotransplanted human breast CSCs aggravated tumor size, and weight as compared with the breast cancer cells, which was effectively perturbed by the combinatorial administration of the potent EGFR inhibitor, **1e** and a low dose of doxorubicin. This signifies that inhibiting EGFR increases the responsiveness of breast CSCs toward existing chemotherapeutics thereby perturbing EGFR‐mediated tumorigenesis.

The epithelial cancer cells are more responsive to chemotherapies. The loss of epithelial phenotype (E‐cadherin) and gain of mesenchymal phenotype (vimentin, N‐cadherin, and fibronectin) results in chemoresistance in breast CSCs due to the intrinsic resistance of EGFR inhibitors to important signal transduction pathways.^51^ During EMT, there is repression of epithelial characteristics and upregulation of mesenchymal markers. The expression of these EMT markers is a prognostic factor for the survival of metastatic breast cancer patients.^51^ The expression of apoptotic proteins, BAX and BCL‐2 in the intrinsic apoptosis pathway is regulated by the activation of JNK and p38.^52^ The literature strongly suggests that JNK and p38 are some of the main prognostic factors in breast cancer.^53^ JNK signaling plays a major role in the suppression of tumorigenesis.^53^ Upregulation of p38 activity causes the apoptotic death of breast cancer cells.^54^ Interestingly, tumor sections in our in vivo studies depicted the combinatorial treatment‐induced activation of the JNK/p38 cascade thereby suggesting an increased apoptosis. Overall, our in vivo observations very well corroborated with the in vitro findings of EGFR inhibition by the potent EGFR inhibitor, **1e** through increasing the efficacy of the doxorubicin against breast CSCs that enhanced EGFR‐driven tumorigenesis.

The major limitation of the present study includes the use of control non‐cancerous epithelial cells of non‐mammary tissue origin. A subsequent evaluation of the safety profile of compound **1e** on sub‐acute toxicity and long‐term exposure of repeated doses would be necessary prior to clinical application.

In conclusion, the inhibition of EGFR‐mediated tumorigenesis of aggressive breast CSCs by the compound **1e** in combination with a low dose of doxorubicin will effectively reduce the tumor growth in TNBCs that provide a therapeutic way forward to reduce the relapse of breast cancer.

## AUTHOR CONTRIBUTIONS

**Trisha Kar:** Investigation (lead); methodology (equal); writing – original draft (equal); writing – review and editing (lead). **Prachi Dugam:** Formal analysis (equal); investigation (equal); methodology (equal); writing – original draft (equal). **Surbhi Shivhare:** Formal analysis (lead); investigation (lead); methodology (equal); writing – original draft (supporting). **Swathi R. Shetty:** Formal analysis (equal); investigation (equal); methodology (equal); writing – original draft (equal). **Subholakshmi Choudhury:** Formal analysis (equal); investigation (equal); methodology (equal); writing – original draft (equal). **Debanjan Sen:** Data curation (equal); formal analysis (lead); investigation (lead); methodology (lead); writing – original draft (lead). **Barnali Deb:** Methodology (supporting). **Swapan Majumdar:** Methodology (supporting); project administration (supporting); supervision (equal). **Sudhan Debnath:** Formal analysis (equal); methodology (equal); project administration (supporting); supervision (equal); writing – original draft (supporting). **Amitava Das:** Conceptualization (lead); formal analysis (lead); funding acquisition (lead); investigation (equal); project administration (lead); resources (lead); software (lead); supervision (lead); writing – original draft (equal); writing – review and editing (lead).

## CONFLICT OF INTEREST STATEMENT

The authors have stated explicitly that there are no conflicts of interest in connection with this article. The authors declare a patent application pending (Application number. 202331017811) on this work.

## ETHICS STATEMENT

All the procedures involving animals followed the guidelines provided by the National ‘Committee for the Purpose of Control and Supervision of Experiments on Animals (CPCSEA)’ and institutional animal ethics committee (IAEC) (approval no. IICT/IAEC/025/2023).

## Supporting information


**Appendix S1:** Supporting Information.

## Data Availability

All the data generated has been included in the manuscript and supplemental data are available online.
