# Supplementary material for: Epidermal growth factor receptor inhibition potentiates chemotherapeutics‐mediated sensitization of metastatic breast cancer stem cells
Source: Cancer Rep (Hoboken). 2024 Mar 24;7(3):e2049. doi: 10.1002/cnr2.2049 (PMC10961089; doi:10.1002/cnr2.2049)
Supplement: Supplementary file 1 — Appendix S1: Supporting Information. [file CNR2-7-e2049-s001.docx]

**EGFR inhibition potentiates chemotherapeutics-mediated sensitization of metastatic breast cancer stem cells**

Trisha Kar^1, 2#^, Prachi Dugam^1#^, Surbhi Shivhare^1, 2^, Swathi R Shetty^1, 2^, Subholakshmi Choudhury^1, 2^, Debanjan Sen^3^, Barnali Deb^4^, Swapan Majumdar^4^, Sudhan Debnath^5^, and Amitava Das^1, 2*^

^1^Department of Applied Biology, Council of Scientific & Industrial Research-Indian Institute of Chemical Technology (CSIR-IICT), Uppal Road, Tarnaka, Hyderabad – 500 007 TS, INDIA.

^2^Academy of Scientific and Innovative Research (AcSIR), Ghaziabad, 201 002, INDIA.

^3^BCDA College of Pharmacy and Technology, Hridaypur, Kolkata – 700 127 WB, INDIA.

^4^Department of Chemistry, Tripura University, Suryamaninagar, 799022, Tripura, INDIA.

^5^Netaji Subhash Mahavidyalaya, Gomati, Udaipur, Tripura, 799114, INDIA.

^#^Authors contributed equally

*Corresponding Author: Dr. Amitava Das, CSIR-IICT, Hyderabad, TS, India. E-mail: [amitavadas@iict.res.in](mailto:amitavadas@iict.res.in) , [amitavadas.iict@gov.in](mailto:amitavadas.iict@gov.in)

Running Title: EGFR-inhibitors mitigate tumorigenesis of metastatic breast CSCs.

*Table ST1. Table representing various human gene-specific primer sequences of CSC markers and apoptotic signaling mediators.*

| Gene | Forward primer sequence | Reverse primer sequence |
| --- | --- | --- |
| *CD24*  *CD44* | 5’ATGTGGCAAGGAAAAACAGG3’  5’AAGGTGGAGCAAACACAACC3’ | 5’TTTGGATGTTGCCTCTCCTT3’  5’AGCTTTTTCTTCTGCCCACA3’ |
| *E-cadherin*  *EpCAM*  *Mucin*  *ALDH1A1* | 5’TGCCCAGAAAATGAAAAAGG3’  5’CGCAGCTCAGGAAGAATGTG3’  5’AGACGTCAGCGTGAGTGATG3’  5'TGTTAGCTGATGCCGACTTG3’ | 5’GTGTATGTGGCAATGCGTTC3’  5’TGAAGTACACTGGCATTGACG3’  5’CAGCTGCCCGTAGTTCTTTC3’  5’TTCTTAGCCCGCTCAACACT3’ |
|  |  |  |
| *Fibronectin*  *N-cadherin* | 5’CAGTGGGAGACCTCGAGAAG3’  5’TTGTATGTTTTCCTTTCAGTGAAGT3’ | 5’CACTGTGACAGCAGGAGCAT3’  5’CTGCCACTTGCCACTTTTCC3’ |
|  |  |  |
| *Vimentin*  *EGFR*  *Eu 18S rRNA* | 5’GGGACCTCTACGAGGAGGAG3’  5’CCTAAGATCCCGTCCATCGC3’  5’AAACGGCTACCACATCCAAG3’ | 5’CGCATTGTCAACATCCTGTC3’  5’GGAGCCCAGCACTTTGATCT3’  5’CCTCCAATGGATCCTCGTTA3’ |

*Table ST2: Table representing various antibodies of apoptotic signaling mediators, CSC markers, and secondary antibodies.*

| Name of antibodies | Source | Company | Catalog no. |
| --- | --- | --- | --- |
| BAX | Rabbit | Puregene | PG-82122 |
| BCL-2 | Rabbit | Novus | NBP267182 |
| Cleaved Caspase 3 | Rabbit | CST | 9664S |
| β-actin | Mouse | Invitrogen | AM4302 |
| GFP | Mouse | CST | 2956S |
| CD44 | Mouse | Abcam | AB-63389 |
| p-EGFR | Rabbit | CST | 3777S |
| p-JNK | Rabbit | CST | 4668S |
| p-P38  Secondary  Secondary | Rabbit  Rabbit  Mouse | CST  CST  CST | 4511S  7074S  7076S |

*Table ST3.*  *Summary of interacting amino acid residues of eleven synthesized and known EGFR inhibitors*

| Sr. No | Ligand | ADV score  kcal/mol | AD Score  kcal/mol | Interacting amino acid residue |
| --- | --- | --- | --- | --- |
| 1 | 1a | ‒8.5 | ‒8.58 | Hydrophobic: Leu-718, Val-726, Lys-745, Met-766, Leu-777, Leu-788, Cys-797, Arg-841, Leu-844, Thr-854, Asp-855 Hydrogen bonding: Gly-719 |
| 2 | 1b | ‒11.5 | ‒10.38 | Hydrophobic: Leu-718, Val-726, Ala-743, Lys-745, Leu-788, Leu-844, Thr-790 Hydrogen bonding: Arg-841 |
| 3 | 1c | ‒10.6 | ‒9.12 | Hydrophobic: Val-726, Ala-743, Lys-745, Leu-844, Thr-790 Hydrogen bonding: Met-793, Arg-841 |
| 4 | 1d | ‒9.9 | ‒9.49 | Hydrophobic: Leu-718, Val-726, Ala-743, Lys-745, Thr-790, Leu-792, Leu-844 Hydrogen bonding: Asn-842 |
| 5 | 1e | ‒10.0 | ‒9.43 | Hydrophobic: Val-726, Ala-743, Lys-745, Leu-844, Thr-790 Hydrogen bonding: Arg-841 |
| 6 | 1f | ‒10.4 | ‒9.65 | Hydrophobic: Leu-718, Val-726, Ala-743, Lys-745, Thr-790, Leu-844, Thr-854 Hydrogen bonding: Arg-841 |
| 7 | 1g | ‒10.3 | ‒10.87 | Hydrophobic: Gly-721, Leu-718, Val-726, Leu-844, Lys-745, Thr-854 Hydrogen bonding: Gly-724, Lys-745 |
| 8 | 1h | ‒8.3 | ‒9.88 | Hydrophobic: Leu-718, Val-726, Ala-743, Lys-745 Leu-788, Thr-790, Leu-792, Leu-844, Phe-997 Hydrogen bonding: Asp-855 |
| 9 | 1i | ‒9.1 | ‒9.92 | Hydrophobic: Leu-718, Val-726, Ala-743, Met-793, Leu-844 Hydrogen bonding: Met-793, Cys-797, Asp-800 |
| 10 | 1j | ‒10.3 | ‒9.13 | Hydrophobic: Val-726, Ala-743, Lys-745, Thr-790. Hydrogen bonding: Met-793, Arg-841 |
| 11 | 1k | ‒12.7 | ‒11.42* | Hydrophobic: Gly-719, Gly-721, Ala-722, Val-726, Ala-743, Lys-745, Thr-790, Leu-844, Arg-84. Hydrogen bonding: Arg-841, Asp-837 |
| 12 | Gefitinib  (DB00317) | ‒7.9 | ‒8.61 | Hydrophobic: Val-726, Lys-745, Thr-790, Leu-844. Hydrogen bonding: Lys-745 |
| 13 | Lapatinib  (DB01259) | ‒8.4 | ‒10.05 | Hydrophobic: Leu-788, Arg-841, Thr-854, Asp-855, Leu-858. Hydrogen bonding: Arg-841 |

*Table ST4.* Dose-response of the six EGFR inhibitors – 1c, 1e, 1g, 1h, 1j, and 1k (1, 2.5, 5, 10 µM) with single conc. of Doxorubicin (0.01 µM) in *MDA-MB-231* and *MDA-MB-468*.

| Sl. No. | Groups | Percent inhibition (%) in  *MDA-MB-231* (Mean ± SEM) | | | | Percent inhibition (%) in  *MDA-MB-468* (Mean ± SEM) | | | |
| --- | --- | --- | --- | --- | --- | --- | --- | --- | --- |
| 1. | Doxo  (0.01 µM) | 21.144 ± 0.701 | | | | 19.142 ± 1.077 | | | |
|  | Compounds | 1 µM | 2.5 µM | 5 µM | 10 µM | 1 µM | 2.5 µM | 5 µM | 10 µM |
| 2. | 0.01 µM Doxo + 1c | 18.35± 1.035 | 20.404±1.004 | 73.731±1.359 | 95.559±0.479 | 20.071±1.864 | 26.632±0.895 | 37.372±0.958 | 97.160±0.957 |
| 3. | 0.01 µM Doxo + 1e | 19.638±1.597 | 54.938±1.363 | 84.179±0.603 | 93.671±0.245 | 17.992±0.703 | 60.206±2.600 | 89.180±1.282 | 93.441±0.935 |
| 4. | 0.01 µM Doxo + 1g | 17.575±0.274 | 22.269±0.621 | 28.886±1.014 | 36.809±0.601 | 14.258±2.345 | 21.582±1.627 | 26.184±0.999 | 39.799±1.596 |
| 5. | 0.01 µM Doxo + 1h | 17.187±0.779 | 20.687±0.866 | 27.403±1.398 | 44.510±0.523 | 20.802±1.206 | 27.130±1.148 | 30.875±1.337 | 42.445±1.320 |
| 6. | 0.01 µM Doxo + 1j | 20.266±1.681 | 24.536±1.327 | 33.672±0.377 | 61.380±0.562 | 23.005±0.701 | 35.759±0.431 | 40.291±1.556 | 70.370±0.580 |
| 7. | 0.01 µM Doxo + 1k | 8.442±0.732 | 24.204±1.027 | 40.184±0.920 | 52.558±0.545 | 23.905±0.482 | 32.595±2.621 | 47.086±1.974 | 51.451±2.700 |

*Figure S1*


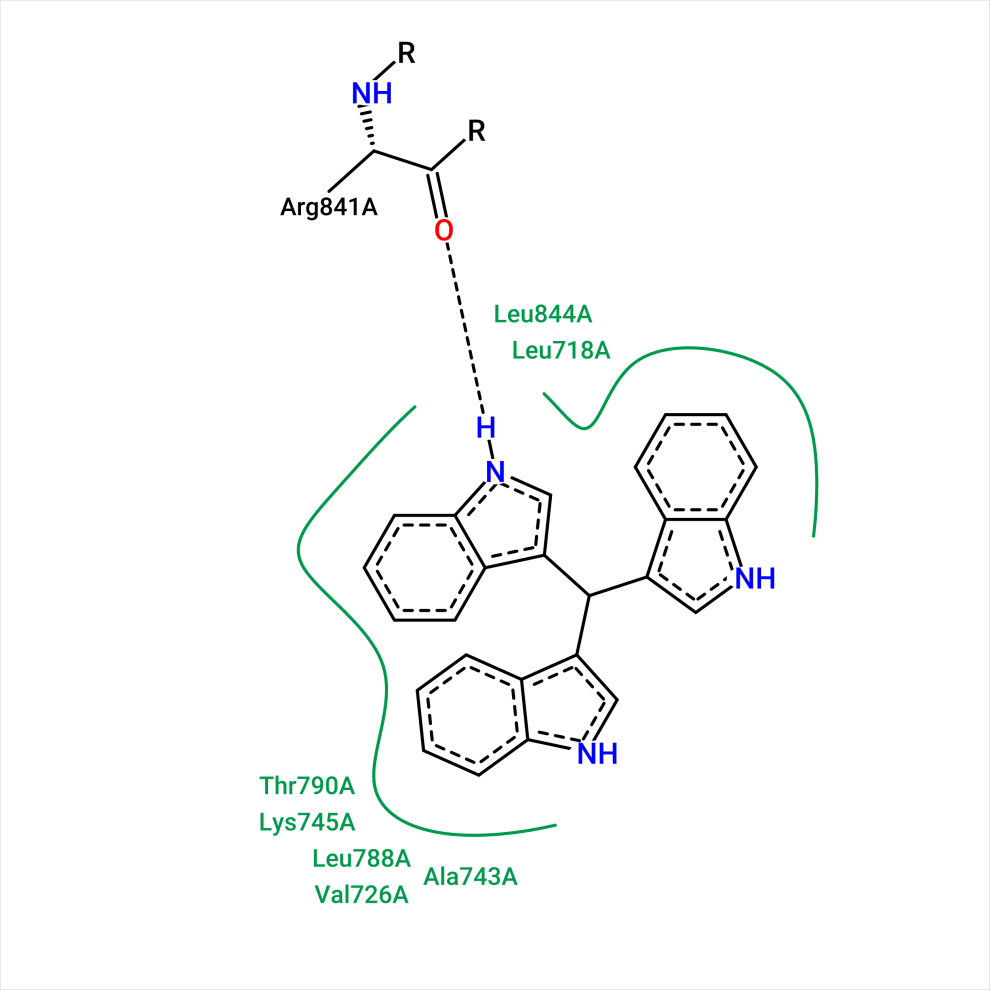


1b


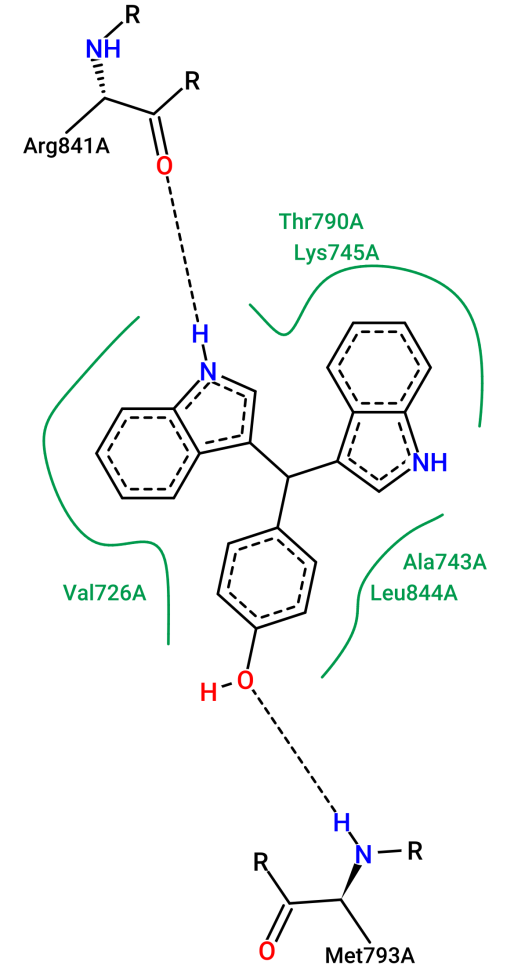

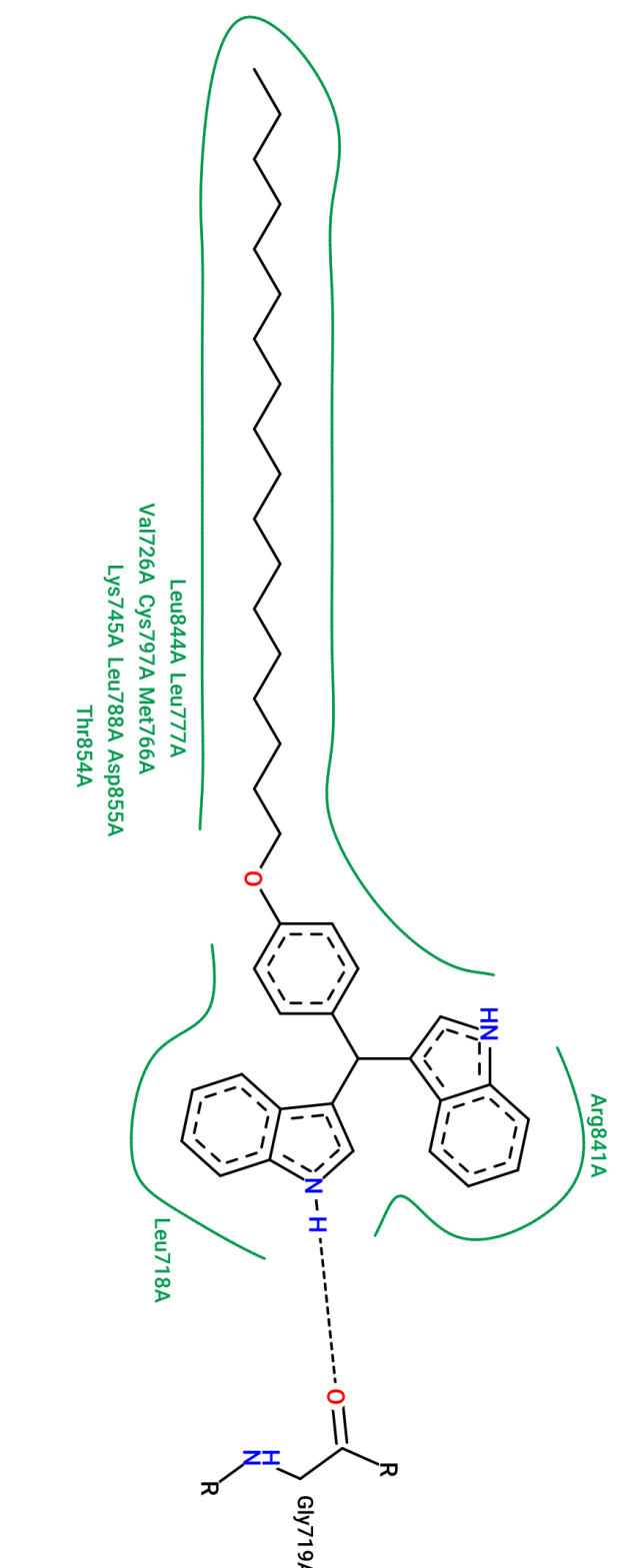


1a


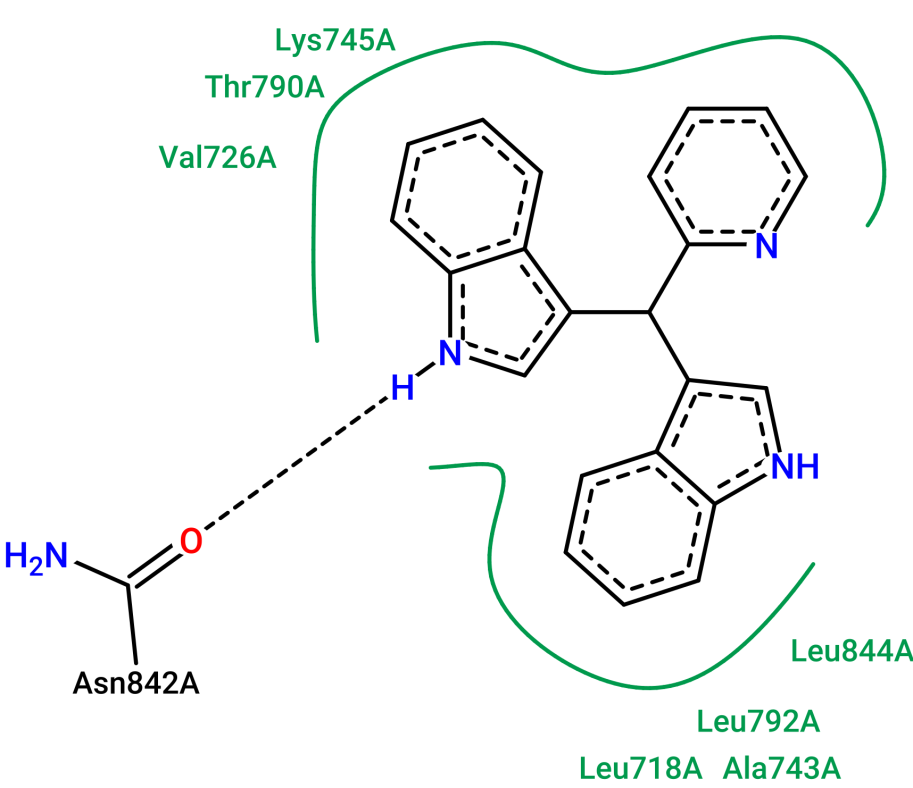


1c


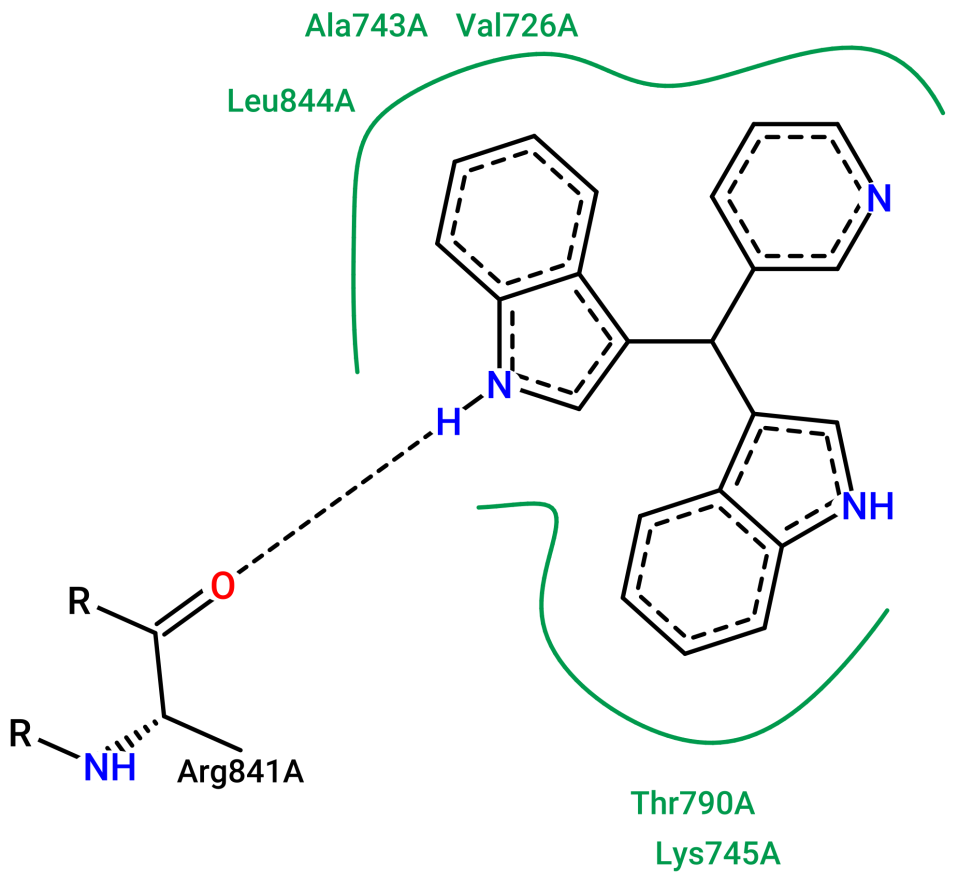


1e


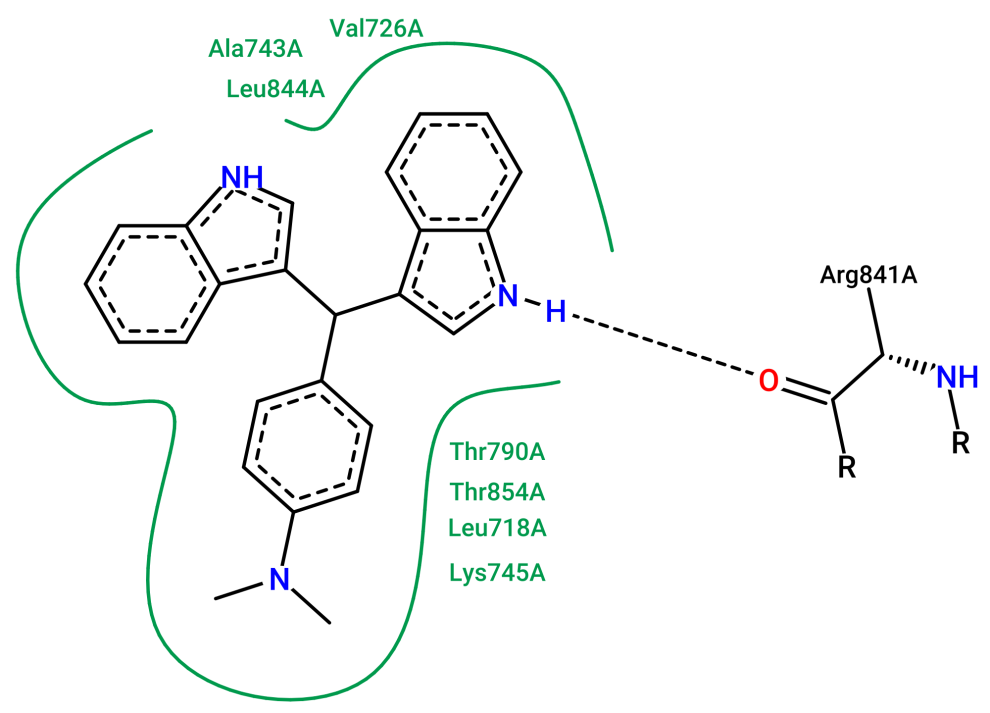


1f


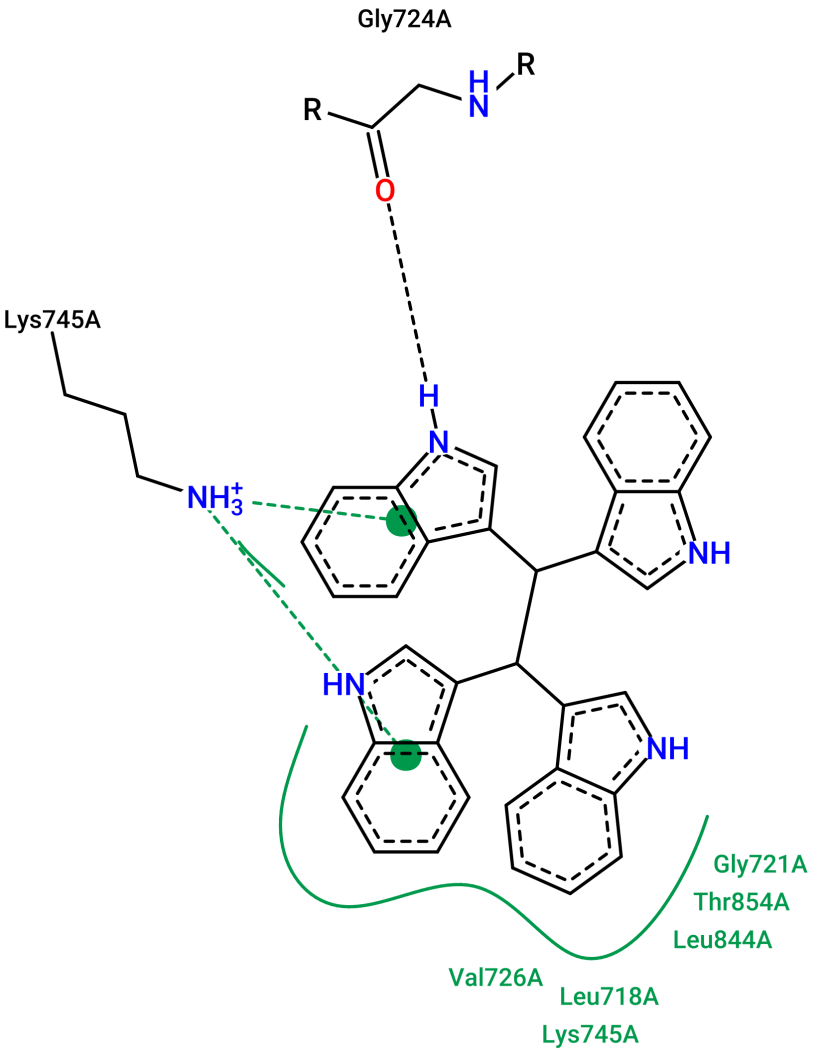

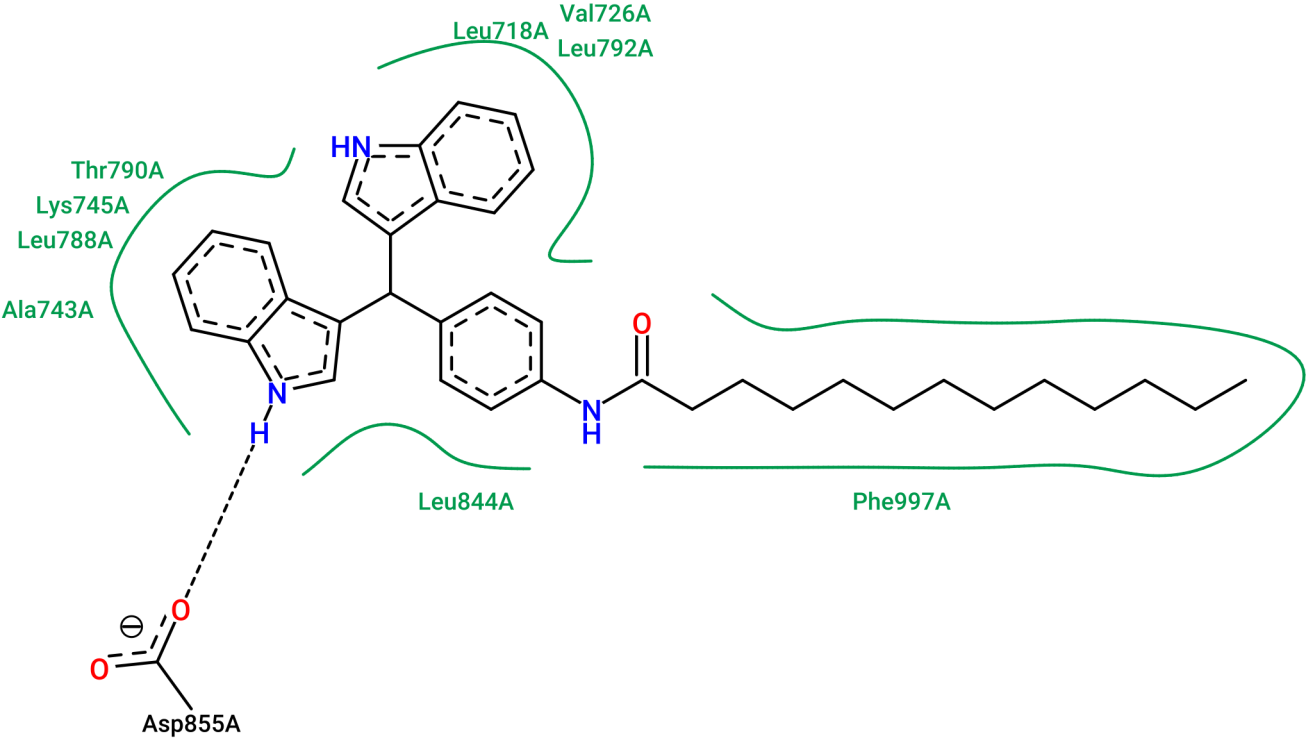


1g

1h


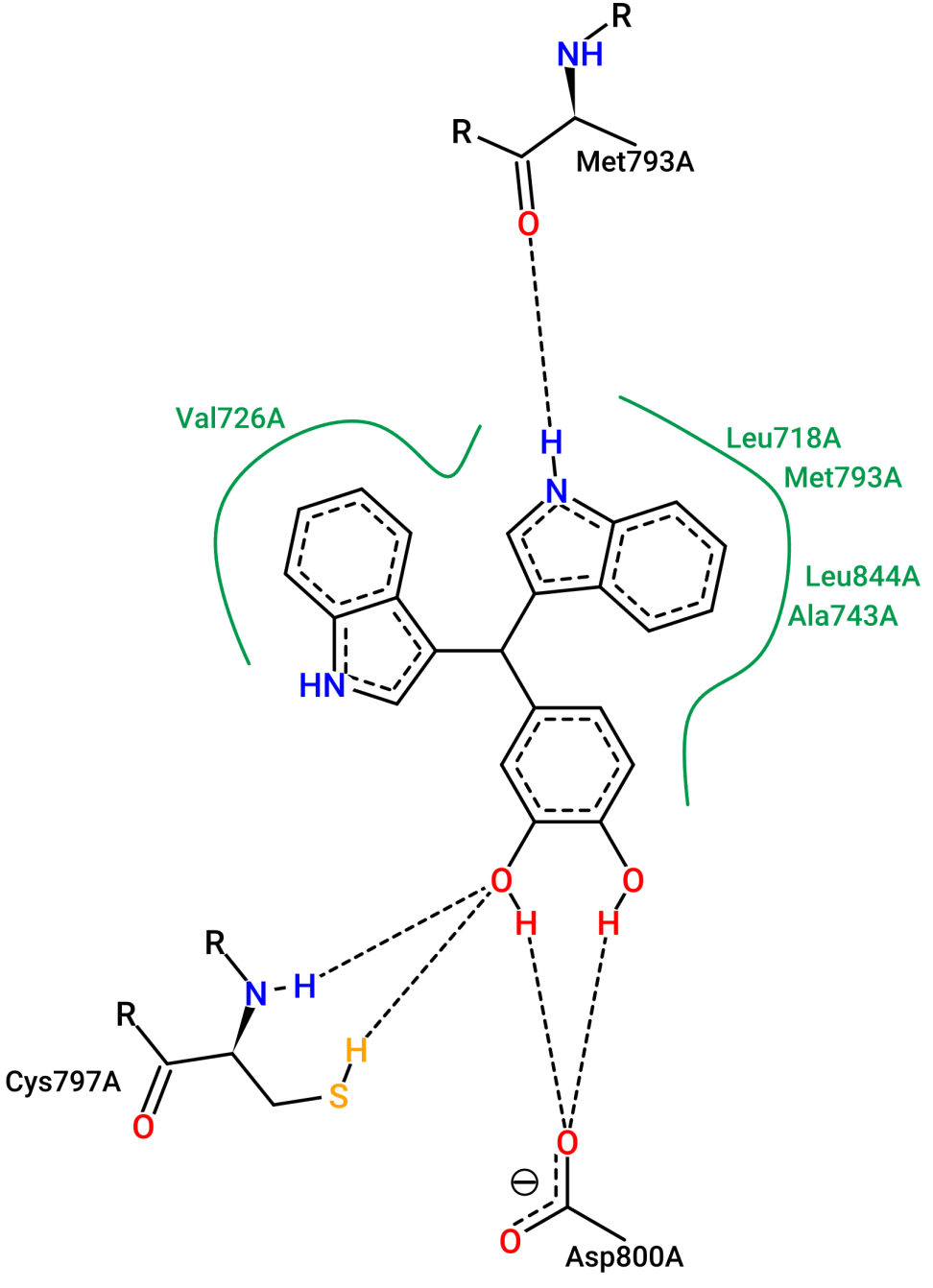

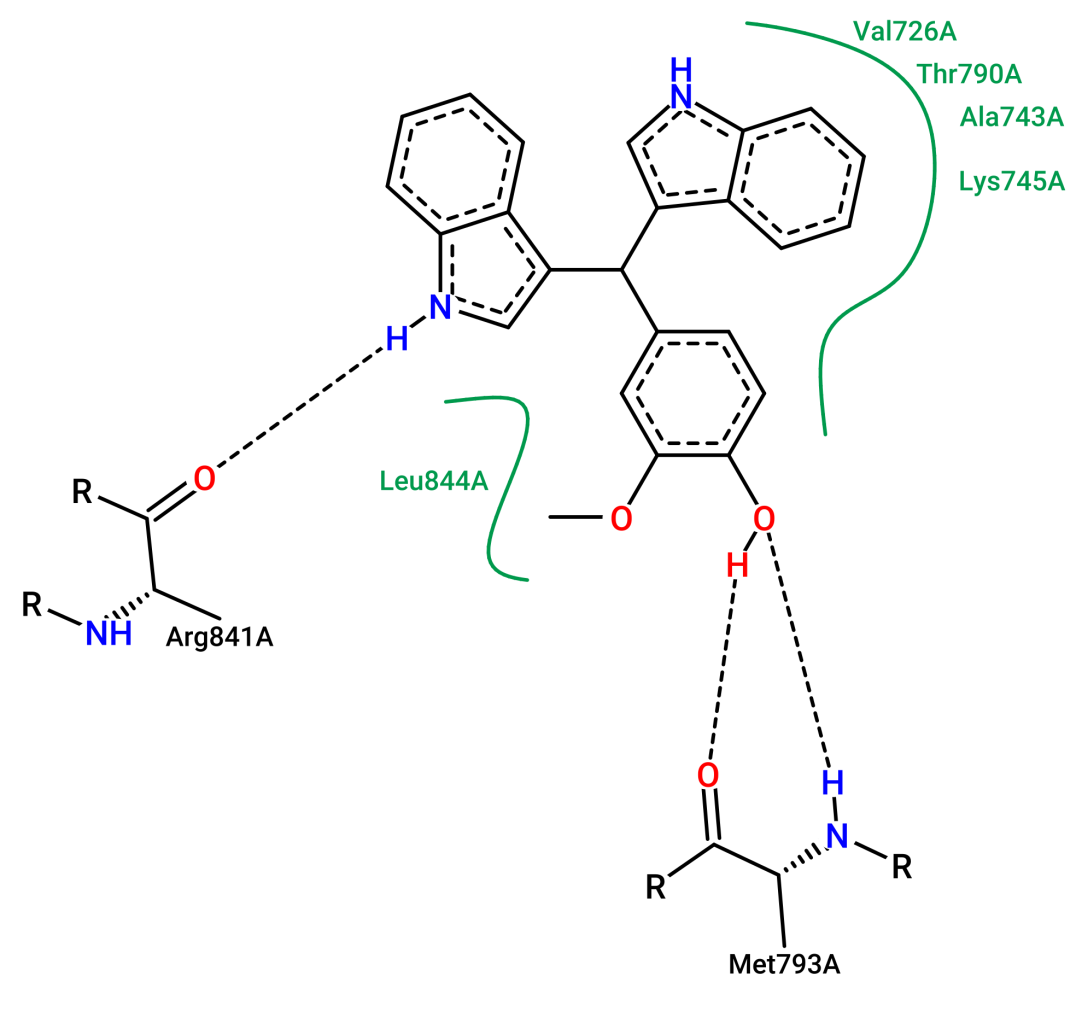


1i

1j


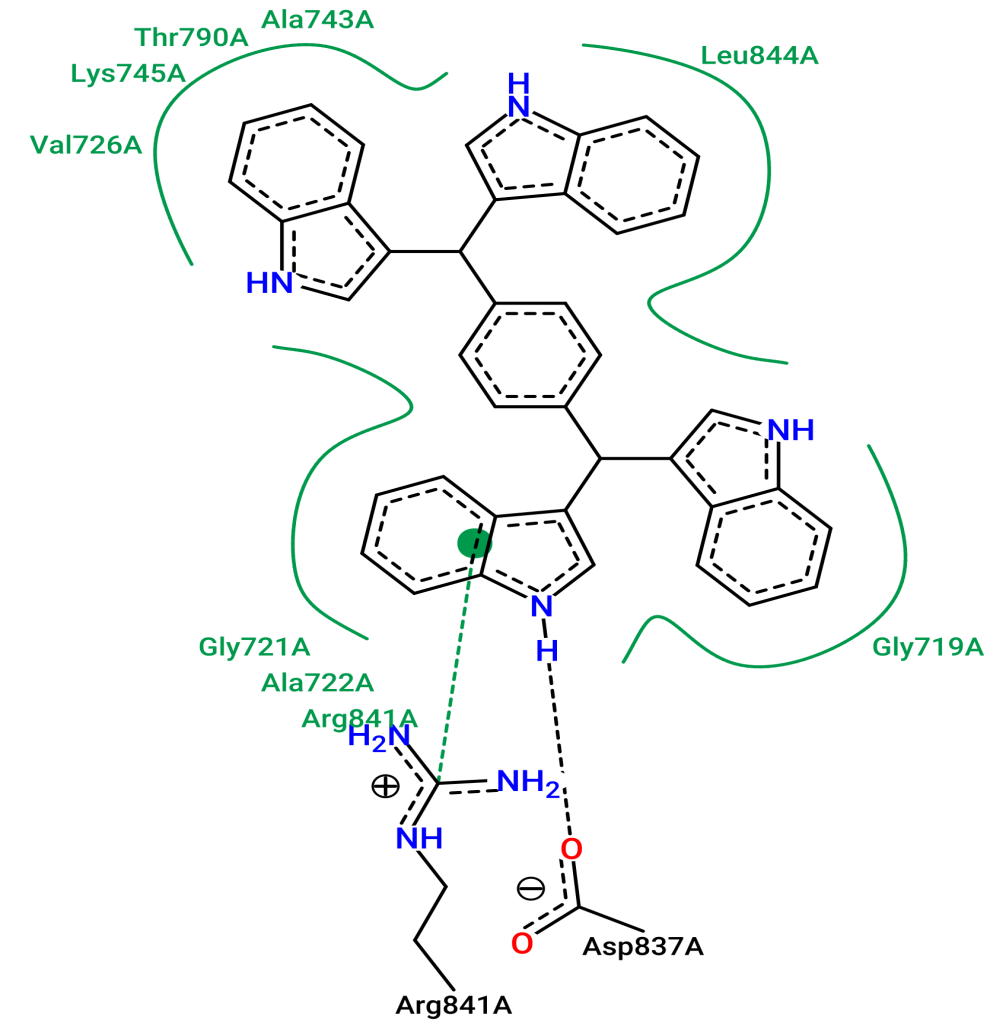


1k


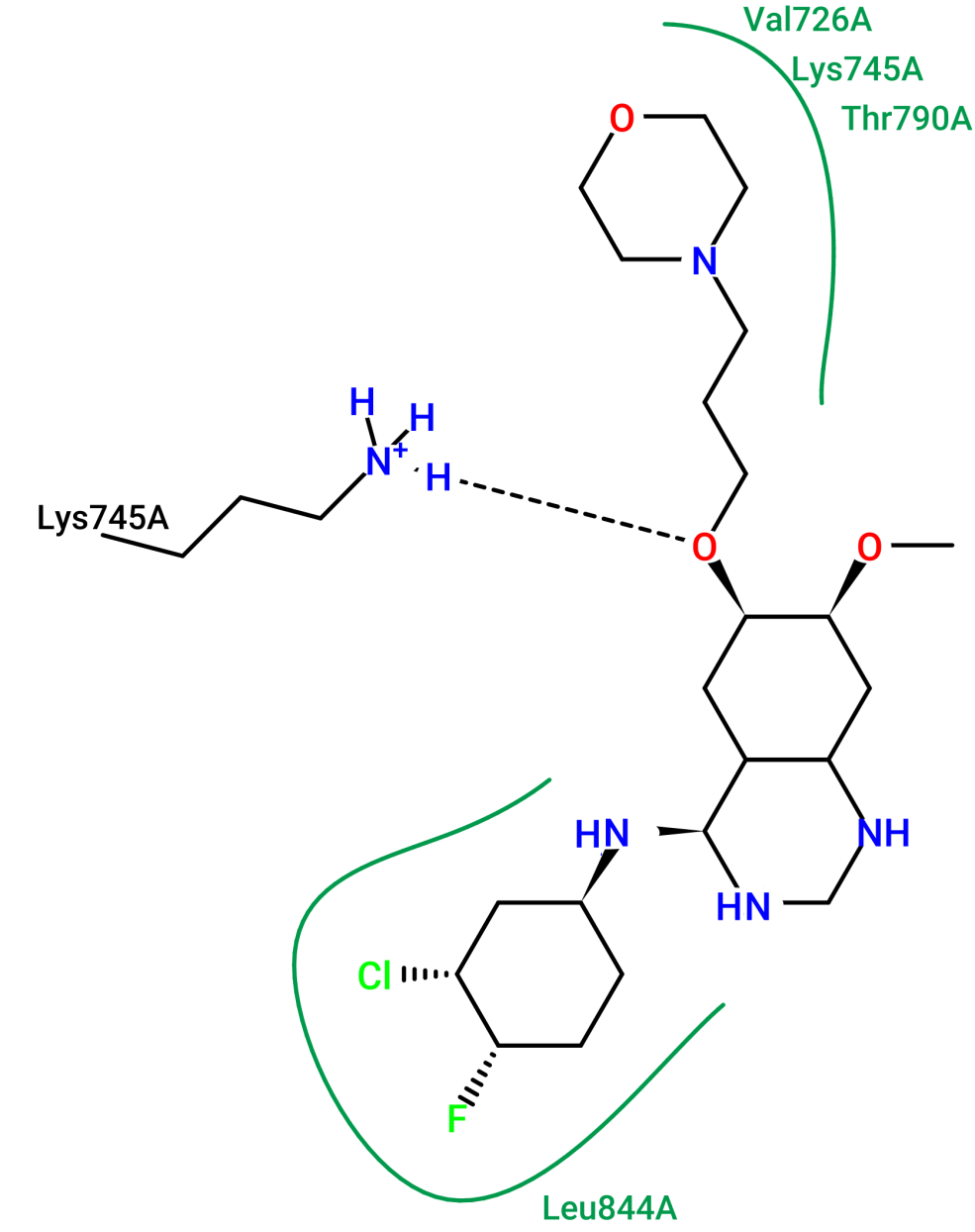

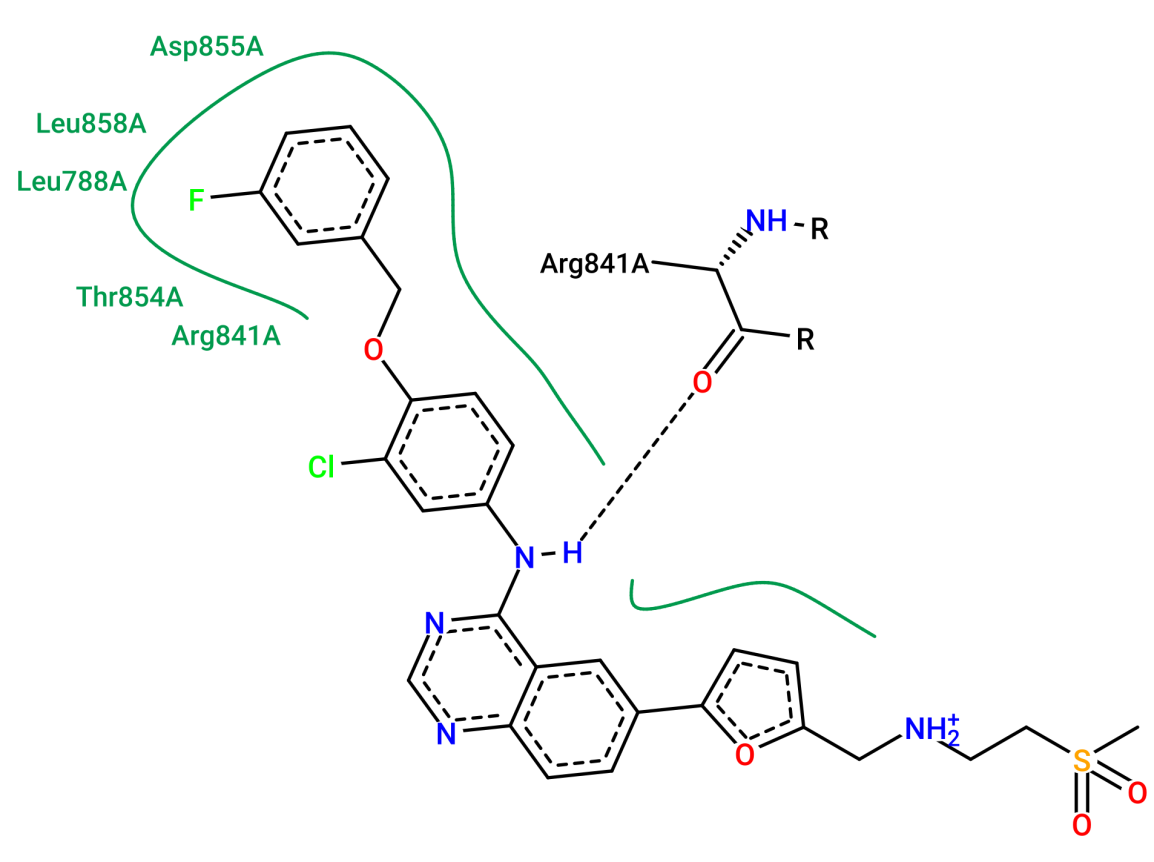


Gefitinib (DB00317)

Lapatinib (DB01259)

1d

*Figure S1. 2-D ligand interaction.* 2-D interaction demonstrating the interaction between the amino acid residues with the EGFR inhibitors/compounds and two commercial EGFR inhibitors - Gefitinib and Lapatinib.

*
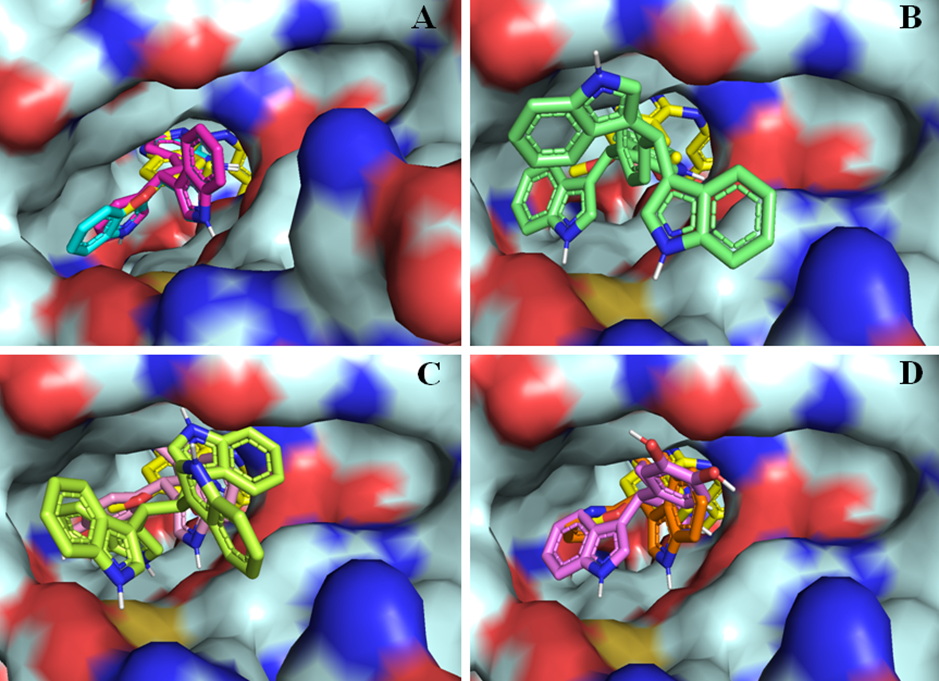
Figure S2*

*Figure S2.* 1b, 1e, and known EGFR inhibitor PD153035 superimposed in the active site and their colors are cyan, magenta, and yellow, respectively. (A) 1k and PD153035 were superimposed, and their colors were lime green, and yellow, respectively; (B) 1d, 1g, and PD153035 were superimposed, and their colors were light pink, lime green, and yellow, respectively; (C) 1d, 1i, and PD153035 and their binding poses were illustrated; (D) 1d, 1i, and PD153035 were superimposed, and their colors were orange, violet and yellow, respectively**.**

*Figure S3*

B

A

*
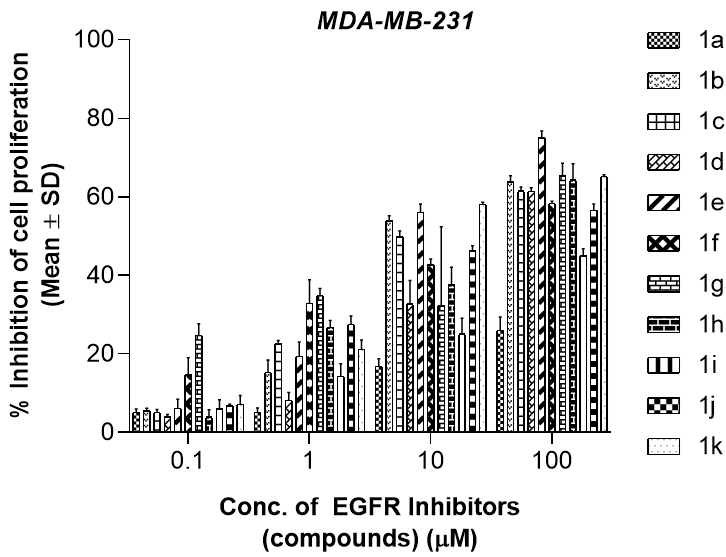

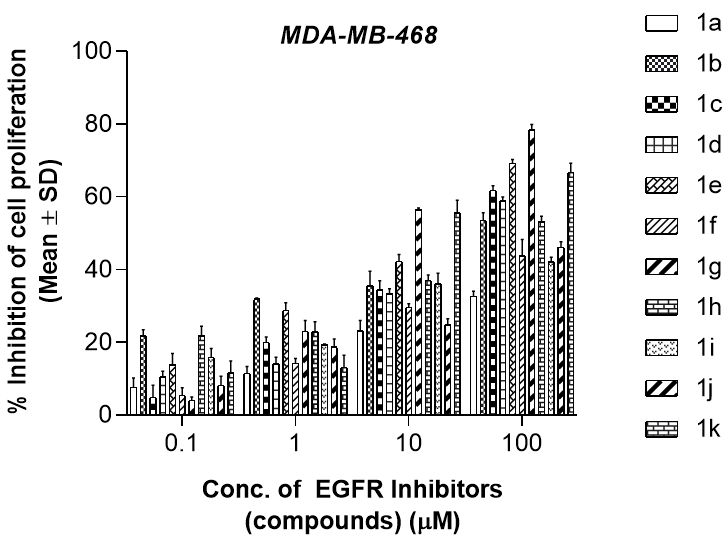
*

C

*Figure S3.* The MTT/Cytotoxicity curves of all the EGFR inhibitors (compounds) in TNBC cell lines – (A) *MDA-MB-231*, (B) *MDA-MB-468*, and (C) control cell line - *HEK293*.

*Figure S4*


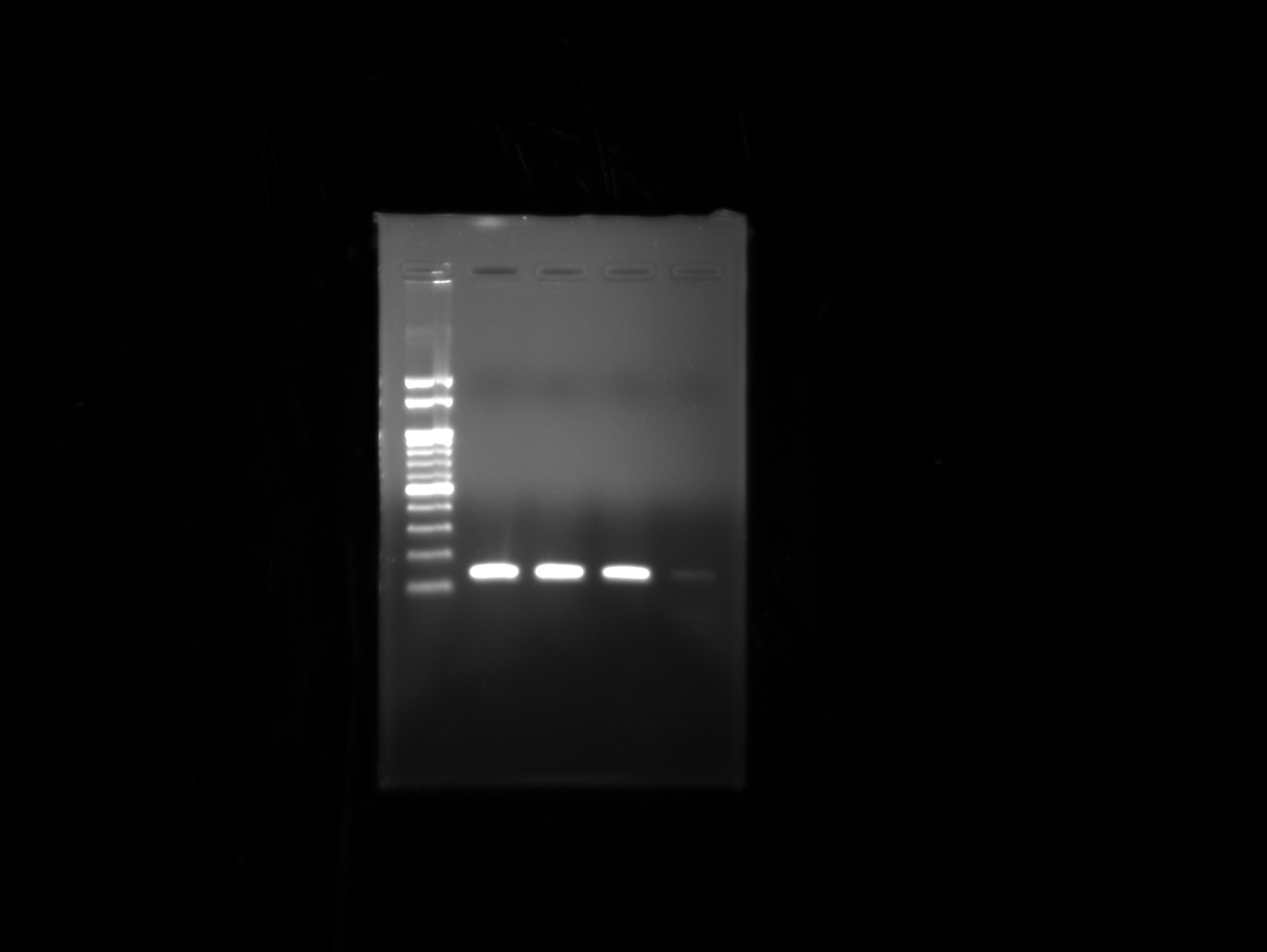

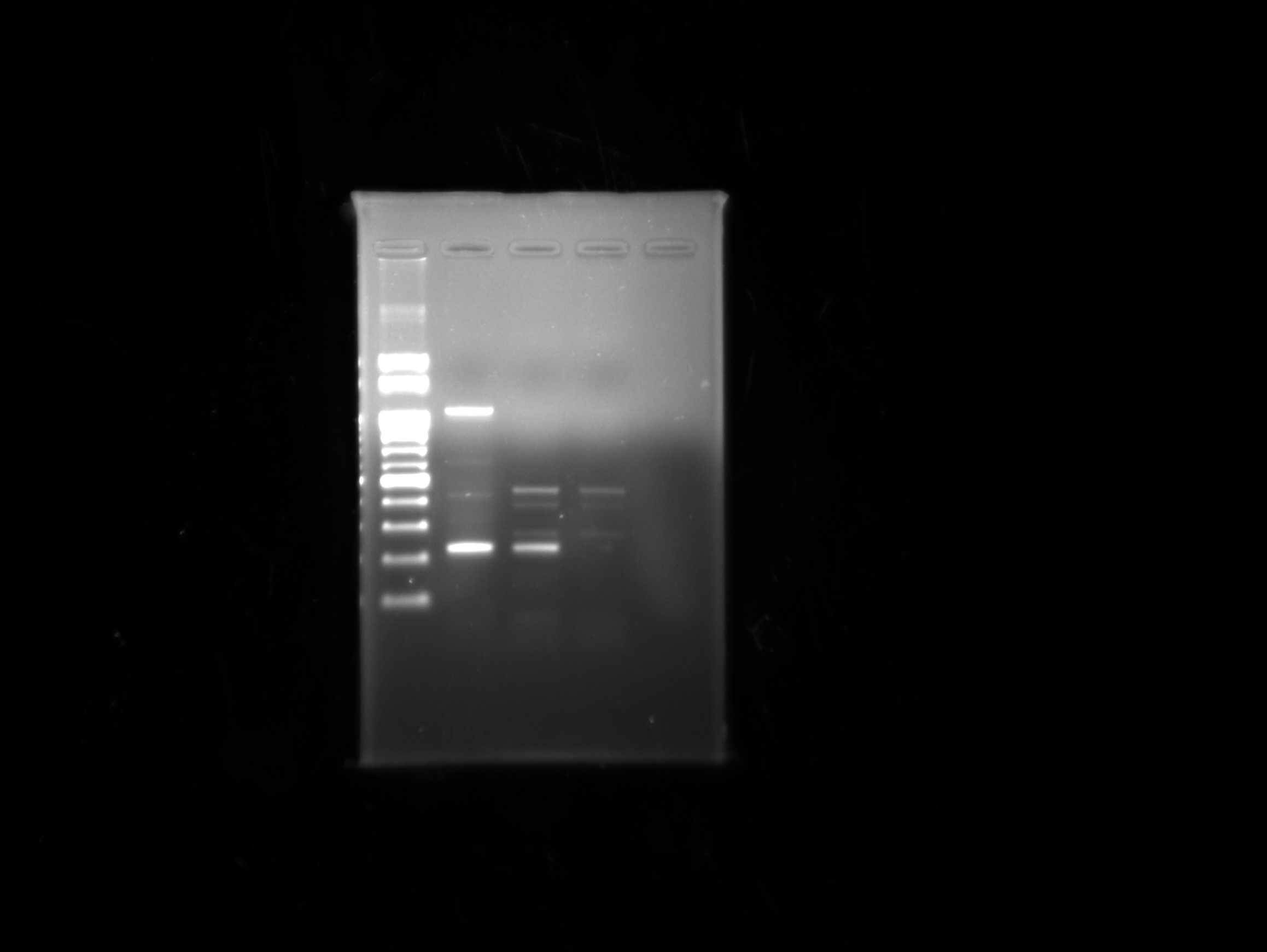


**Eu 18S rRNA**

**EGFR**

**240**

**155**

**Base pair**

**(bp)**

**MDA-MB-231**

**MDA-MB-468**

**HEK-293**

*Figure S4.* Differential endogenous mRNA expression of EGFR in TNBC cells - *MDA-MB-231*, *MDA-MB-468,* and control cells - *HEK293*.

*Figure S5*

*Figure S5.* Dose-response of doxorubicin (0.01, 0.1, 1.0 µM) with single conc. of potential EGFR inhibitors (5 µM) in (A) *MDA-MB-231* and (B) *MDA-MB-468* (Combination of drug regimens of doxorubicin with all the EGFR inhibitors.

*Figure S6*

A

*
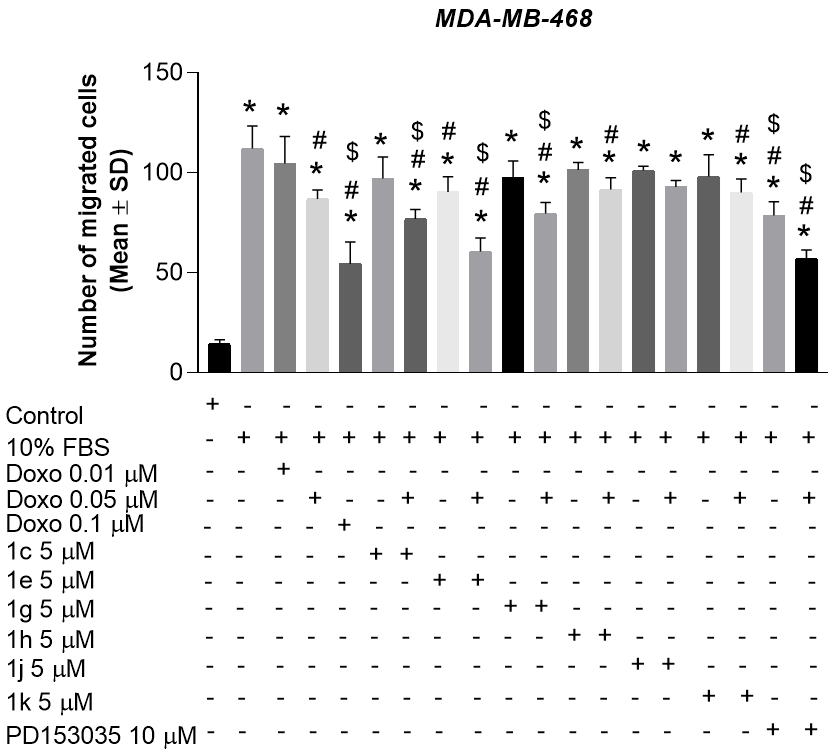

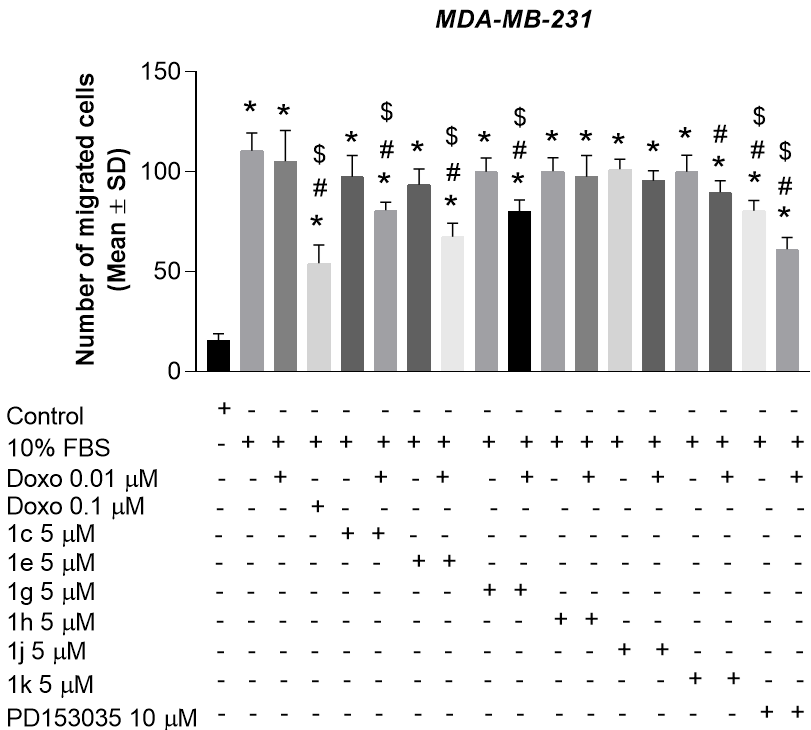
*

B

*Figure S6*. Graphs depicting the migratory potential of TNBC cell lines (A) *MDA-MB-231* and (B) *MDA-MB-468* in the presence of combinatorial treatment of Doxorubicin with potential EGFR inhibitors (compounds - 1c, 1e, 1g, 1h, 1j, and 1k) or a commercial EGFR inhibitor, PD153035. Data represents the number of cells migrated as Mean ± SD of three independent experiments. p≤0.05 as compared to *control, ^#^10% FBS group, and ^$^Doxo 0.01 µM and 0.05 µM in *MDA-MB-231* and *MDA-MB-468* respectively.

*Figure S7*

*MDA-MB-231*

*
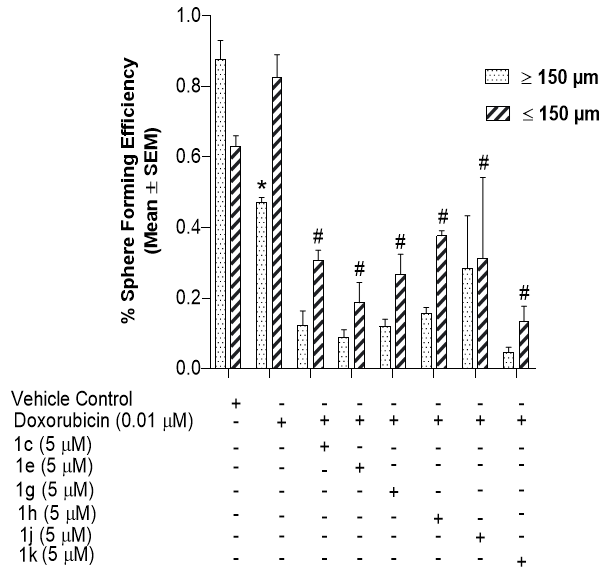

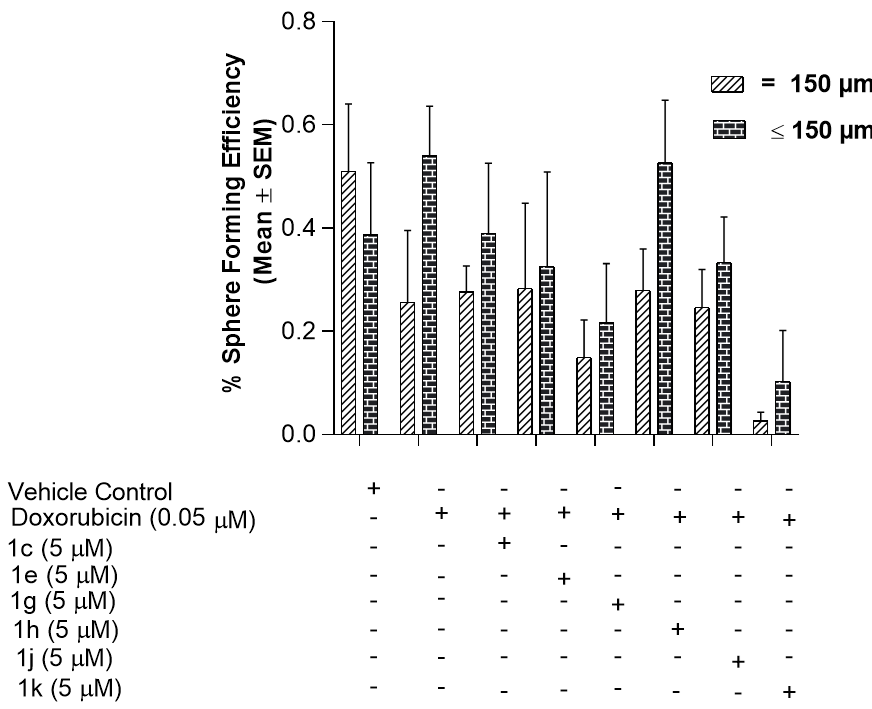
*

*MDA-MB-468*

B

A

*Figure S7.* *EGFR inhibition modulates the mammosphere formation efficiency of breast cancer cells****.*** Quantification of the mammosphere images of the *MDA-MB-231* and *MDA-MB-468* TNBC cell lines. (A) *MDA-MB-231* demonstrated a significant lowering in the percent of sphere-forming efficiency upon combinatorial treatment with doxorubicin below its IC50 concentrations (0.01 µM) and designed EGFR inhibitors at 5 µM concentration. (B) Another TNBC cell line-*MDA-MB-468* showed a similar trend of lowering in the sphere-forming capabilities with the treatment of doxorubicin at 0.05 µM (below IC50 value in *MDA-MB-468*) and designed EGFR inhibitors (1c,1e,1g,1h,1j and 1k) at 5 µM concentration. Statistical significance was determined from experiments, performed in triplicates (n=3), p <0.05 (2-way ANOVA) as compared to *control and ^#^Doxorubicin (0.01 µM).

*
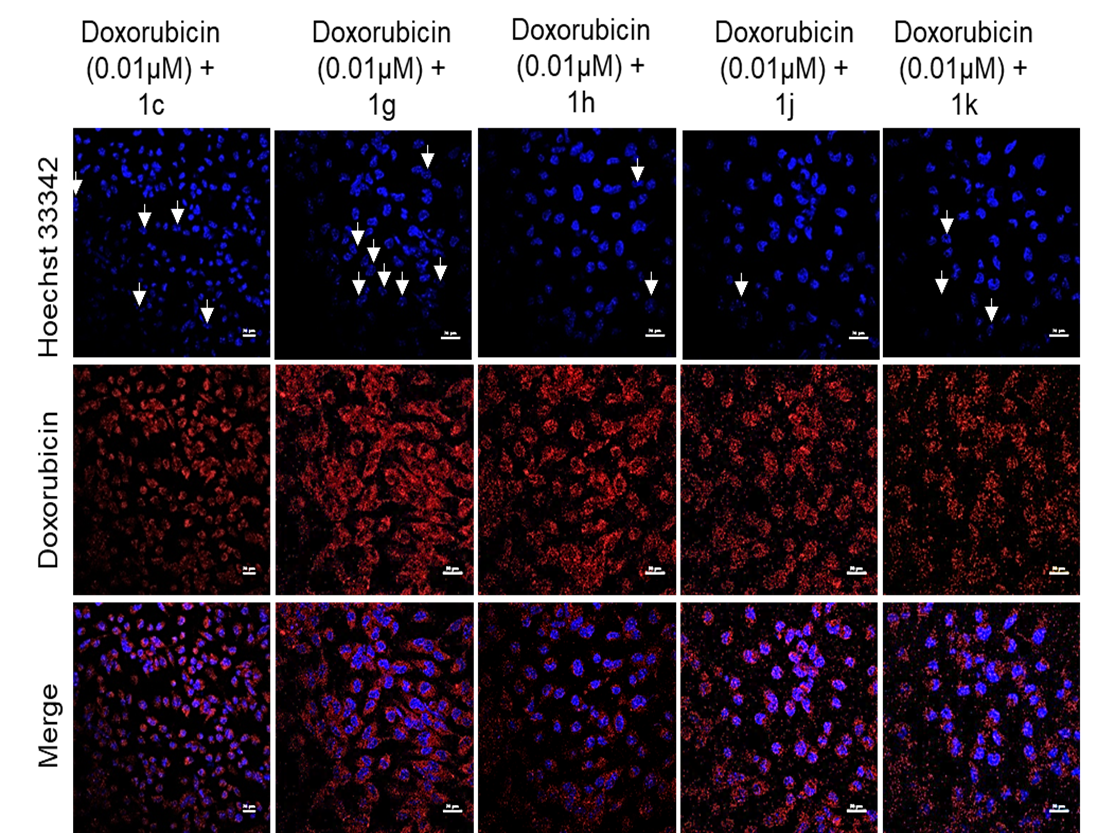
Figure S8*

*Figure S8. Detection of apoptosis induced by the combinatorial treatment of doxorubicin and selected compounds/EGFR inhibitors.* Representative images depicting the apoptosis of *MDA-MB-231* cells, treated with a combination of doxorubicin (0.01 µM) and compound 1c, 1g, 1h, 1j, and 1k (5 µM) for 48 h and subjected to staining with Hoechst 33342*.*

*Figure S9*

D

C

A

B


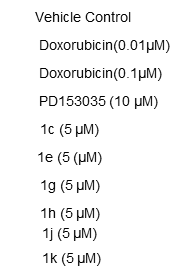

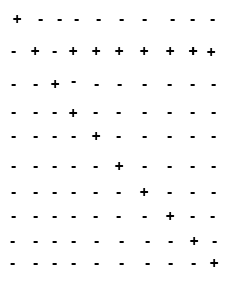

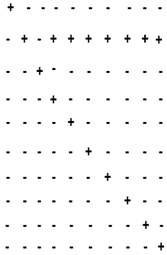

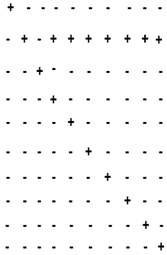

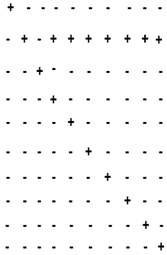


*Figure S9. Graphs showing the densitometric analysis of western blot images.* Quantitative expression of BCL2, BAX, BAX/BCL2 ratio, and Cytochrome-C. Compound **1e** depicted a marked decrease in the BCL2 levels, elevation in BAX protein level, and increase in BAX/BCL2 ratio in *MDA-MB-231* cells.

*Figure S10*


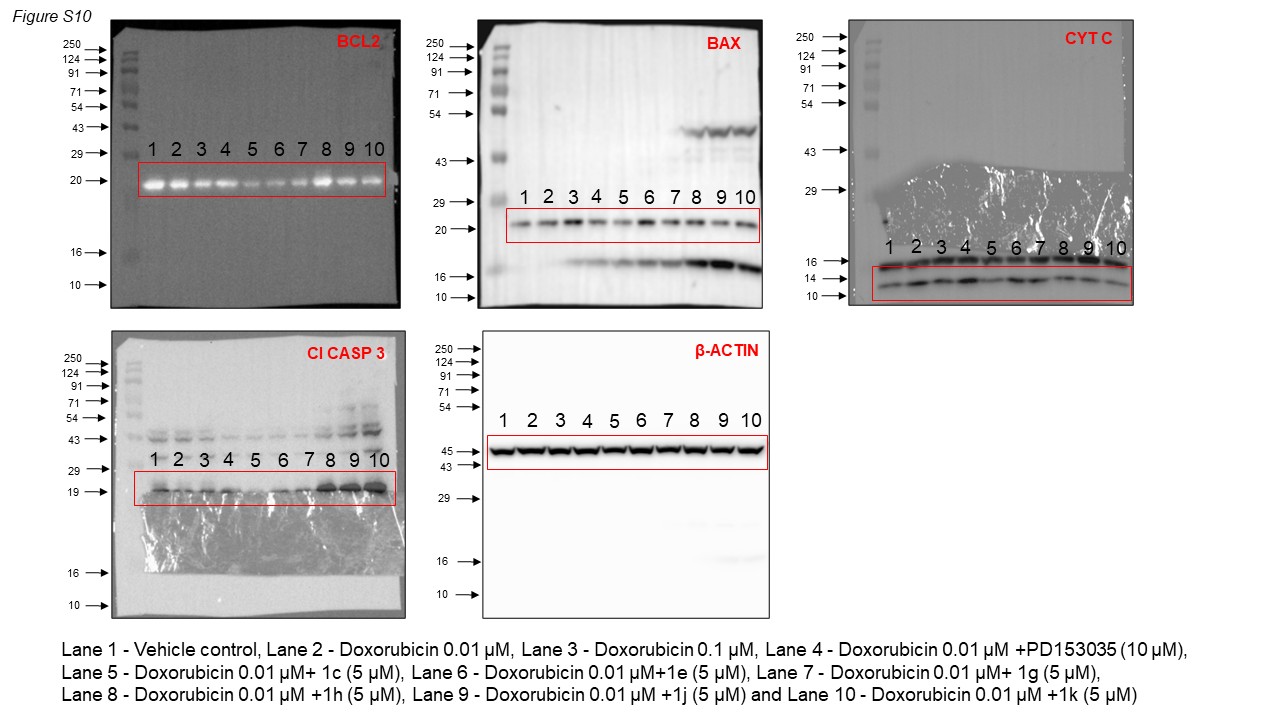


*Figure S10. Graph depicting the full blots of western blot images.* Raw files of the western blot images included in the main figure 2C.

**************************************************************************************************
